# Towards a Rigorous Network of Protein-Protein Interactions of the Model Sulfate Reducer *Desulfovibrio vulgaris* Hildenborough

**DOI:** 10.1371/journal.pone.0021470

**Published:** 2011-06-28

**Authors:** Swapnil R. Chhabra, Marcin P. Joachimiak, Christopher J. Petzold, Grant M. Zane, Morgan N. Price, Sonia A. Reveco, Veronica Fok, Alyssa R. Johanson, Tanveer S. Batth, Mary Singer, John-Marc Chandonia, Dominique Joyner, Terry C. Hazen, Adam P. Arkin, Judy D. Wall, Anup K. Singh, Jay D. Keasling

**Affiliations:** 1 Virtual Institute of Microbial Stress and Survival, Lawrence Berkeley National Laboratory, Berkeley, California, United States of America; 2 Physical Biosciences Division, Lawrence Berkeley National Laboratory, Berkeley, California, United States of America; 3 Earth Sciences Division, Lawrence Berkeley National Laboratory, Berkeley, California, United States of America; 4 Life Sciences Division, Lawrence Berkeley National Laboratory, Berkeley, California, United States of America; 5 Department of Biochemistry, University of Missouri, Columbia, Missouri, United States of America; 6 Department of Molecular Microbiology and Immunology, University of Missouri, Columbia, Missouri, United States of America; 7 Biosystems Research Department, Sandia National Laboratory, Livermore, California, United States of America; 8 Department of Chemical Engineering, University of California, Berkeley, California, United States of America; 9 Department of Bioengineering, University of California, Berkeley, California, United States of America; 10 Joint BioEnergy Institute, Emeryville, California, United States of America; The Research Institute for Children, United States of America

## Abstract

Protein–protein interactions offer an insight into cellular processes beyond what may be obtained by the quantitative functional genomics tools of proteomics and transcriptomics. The aforementioned tools have been extensively applied to study *Escherichia coli* and other aerobes and more recently to study the stress response behavior of *Desulfovibrio vulgaris* Hildenborough, a model obligate anaerobe and sulfate reducer and the subject of this study. Here we carried out affinity purification followed by mass spectrometry to reconstruct an interaction network among 12 chromosomally encoded bait and 90 prey proteins based on 134 bait-prey interactions identified to be of high confidence. Protein-protein interaction data are often plagued by the lack of adequate controls and replication analyses necessary to assess confidence in the results, including identification of potential false positives. We addressed these issues through the use of biological replication, exponentially modified protein abundance indices, results from an experimental negative control, and a statistical test to assign confidence to each putative interacting pair applicable to small interaction data studies. We discuss the biological significance of metabolic features of *D. vulgaris* revealed by these protein-protein interaction data and the observed protein modifications. These include the distinct role of the putative carbon monoxide-induced hydrogenase, unique electron transfer routes associated with different oxidoreductases, and the possible role of methylation in regulating sulfate reduction.

## Introduction

Recent functional genomics efforts have established *Desulfovibrio vulgaris* Hildenborough as a model anaerobe. Much of the information currently available on this sulfate reducer is based on quantitative transcriptomics analyses of stress response behavior [1], [2], [3], [4], [5], [6]. Identification of protein-protein interaction networks in an organism complements information that can be gleaned from other functional genomics approaches for the purpose of building system and cellular models. While several approaches exist for identifying protein-protein interactions [7], two that have recently gained popularity include the exogenous and endogenous ‘pull-down’ methods [8], [9], [10]. The exogenous method consists of immobilizing heterologously expressed bait proteins and incubating them with whole cell lysate of the organism under investigation. In this case the tagged bait competes for the same set of interacting partners already associated with its native counterpart in the cell lysate, hence identification of the interaction network is dependent on the relative protein concentrations and the inherent dissociation constants of the endogenous protein and other partners in the native complex(es). As a result exogenous pull-down methods can lead to a large number of false positives originating from non-specific interactions detected due to the excess of immobilized bait in relation to the interacting partners from the native complex. While this approach is amenable to a high throughput scale-up, its utility for reliably detecting interactions with a high degree of coverage and observing dynamic interactions under different cellular states is limited.

Many of the problems with the exogenous approach can be alleviated using an endogenous approach that relies on chromosomal modification of the organism under investigation to incorporate an affinity tag at either the amino- or carboxy-ends of the protein of interest. This approach relies on native or near-native concentrations of interacting partners and assumes that the intact and functional complex consisting of the affinity-tagged bait and prey proteins can be recovered provided the tag does not interfere with complex formation. The endogenous approach requires well-functioning genetic tools for chromosomal modification and to some extent depends on the cellular concentrations and compartmentalization of the bait protein. Large-scale protein-protein interaction datasets generated with this approach have been reported for *Saccharomyces cerevisiae* [11], [12] and *Escherichia coli* K12 [10], [13].

**Figure 1:**
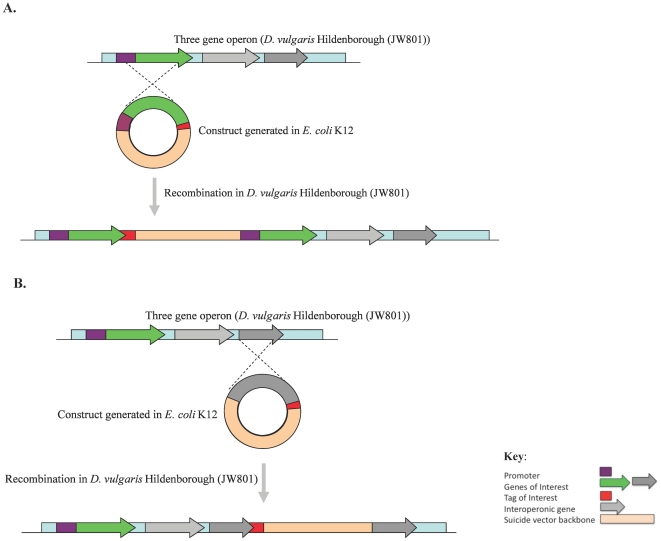
The single cross-over strategy for tagged mutant generation. **A.** Tagging the first member of a three-gene operon. **B.** Tagging the last member of a three-gene operon.

In this paper we describe our efforts to apply the endogenous ‘pull-down’ approach for identifying protein-protein interactions in the sulfate reducer *D. vulgaris*. Our approach is based on suicide-vector-assisted chromosomal tagging (Fig. 1). We appended an eight amino-acid tag (*Strep*-tag II; IBA, St. Louis, MO) to the C- terminus of twelve proteins from various functional categories. The *Strep*-tag approach offers the simplicity and convenience of a single-step method and has been touted for its reliability and efficacy in high throughput applications [14], [15]. In a systematic comparison of eight elutable affinity tags (hexahistidine (HIS), calmodulin-binding peptide (CBP), covalent yet dissociable NorpD peptide (CYD), FLAG, heavy chain of protein C (HPC), glutathione S-transferase (GST), maltose-binding protein (MBP) and *Strep*-tag II), the latter (*Strep*-tag II) was reported to possess the ideal combination of excellent purification with good yields at a moderate cost [16]. The *Strep*-tag II has been successfully employed for purifying functional holoenzyme protein complexes from mammalian cells [14]. The interacting proteins reported in the aforementioned study were found to be identical to those identified using tandem affinity purification experiments for the same baits. The *Strep*-tag method does not require the availability of cofactors or the coexpression of modifying enzymes and is unlikely to interfere with complex functionality given its small size thus making it suitable for use in generic protein-protein interaction studies in a variety of cell types [14]. Baits chosen in the current study fell into two groups: highly conserved proteins with known interacting partners in *E. coli* or proteins unique to *D. vulgaris* energy metabolism. The interacting partners associated with these *Strep-*tagged baits were identified in replicate using affinity purification followed by liquid chromatography-mass spectrometry (LC-MS)-based analyses.

To date only a single study has reported a protein-protein interaction network based on replicate pull down experiments [17]. Moreover, no previously reported protein-protein interaction networks have directly incorporated protein-protein interaction confidence based on experimental observations. In the present study, we combined the use of biological replicates, exponentially modified Protein Abundance Index (emPAI) scores [18], and control pull-down experiments with no tagged bait protein to assign confidence to bait-prey observations. While it has been established that MS instruments have a high degree of reproducibility for identifying peptides when repeatedly analyzing the same sample (i.e. technical replicates), this consistency remains untested in the biological replicate setting where samples are derived from independent cell cultures and when different purification columns are used. The use of biological replicates, albeit expensive and time-consuming, enables identification of stochastic errors, an important source of false positives in protein-protein interaction data [19]. To analyze the replicate pull-down experiments we devised a computational method to transform the raw LC-MS replicate data into associations between bait and prey proteins. We corrected for nonspecific interactions, i.e. false positives stemming from systematic errors, by incorporating results from pull-down experiments with no tagged bait present as a negative control. The resulting pseudo-confidence scores allowed us to identify a confident subset of the data, where all interactions where observed in triplicate and above the level, or in special cases equal to, that observed in the control. To assess statistical significance for each putative interacting bait-prey pair we performed a bootstrap test. To make the test more applicable to small interaction datasets, we also sampled from bait pull down replicate data dissimilar from the bait in question to obtain additional samples of the control data. We used a high confidence subset of bait-prey interactions to reconstruct a partial network of protein-protein interactions for *D. vulgaris*. We validated this network with a series of comparative and functional genomics analyses and statistical tests, and discuss the biological significance of the observations.

## Results and Discussion

In this study we engineered mutant strains of JW801 (Fig. 1) to encode *Strep-*tagged bait proteins for identification of potential interacting partners. JW801, a strain of *D. vulgaris* Hildenborough lacking the native plasmid, pDV1 (202 kb, 157 ORFs), was chosen because of its increased transformation efficiency relative to the wild type strain when transformed with *E. coli* K12-derived plasmids. The lack of pDV1 results in the inability of JW801 to fix nitrogen and slows its growth on LS4D medium; however, pDV1 encodes no essential functions under laboratory conditions.

**Figure 2:**
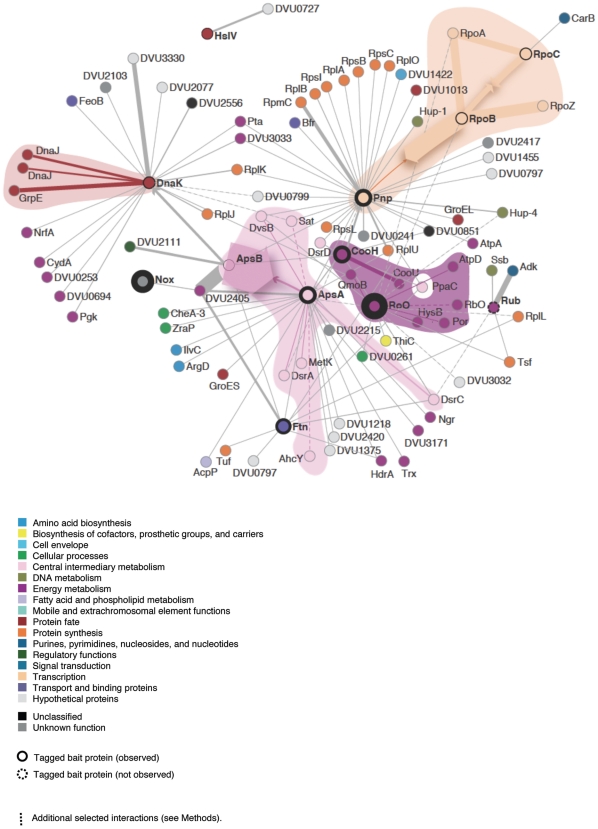
Partial *D. vulgaris* Hildenborough (JW801) high confidence protein-protein interaction network. Shown are the high confidence bait-prey protein interaction pairs from this study. Edges connecting nodes indicate a detected high confidence interaction between a bait and a pulled down prey protein. Nodes in the network are colored by TIGR functional role, as are edges where both nodes belong to the same TIGR role. Bait protein nodes are surrounded by a thicker black circle proportional to the normalized adjusted median-max emPAI value for the bait protein. The dotted node indicates the bait, which was not observed (Rub). Dotted edges indicate interactions with a median-max emPAI value equal to the control but where the bait was also observed in the control with a high emPAI value (see Methods). Interconnected sets of nodes belonging to the same TIGR role are shaded with a lighter hue of the TIGR functional role color. Head-on arrows indicate reciprocally detected interactions and the width of the arrow corresponds to the normalized adjusted median-max emPAI value for the prey protein. Interactions corresponding to p<0.001 from bootstrap analysis are shown in Table S12.

The *Strep*-tag II sequence was appended to all genes discussed in this study at the 3′ end. The *Strep*-tag II [20], [21] is an eight-amino acid peptide (WSHPQFEK) that binds with strong selectivity to an engineered version of streptavidin called *Strep*-Tactin (K_d_ = 1 µM) and has been used previously for the identification of protein-protein interactions [14]. Predicted operon structures [22], TIGR functional roles [23], and other properties for bait proteins chosen in this study are shown in Table S1. The JW801 protein-protein interaction data in this study were composed of 134 protein-protein interactions arising from 12 bait proteins (Fig. 2). In the following sections we report results of the interaction network reliability, compare interactions for highly conserved baits, and discuss the biological implications of bait-prey interactions specific to *D. vulgaris*.

### Data quality analysis and validation

Bait-prey interactions described in this study may be divided into two groups. The first group is composed of tagged-bait proteins and their true interacting partners. The second group consists of false positive interactions: ‘sticky’ proteins bound to the bait pull-down column either due to their inherent abundance or some affinity for *Strep*-Tactin as well as other proteins that interact with these ‘sticky’ proteins. For large protein interaction datasets false positives may be estimated empirically from the protein interaction dataset by measuring the promiscuity of prey proteins [10], [13]. However, for smaller datasets these methods are not applicable due to the limited data available for producing estimates. Instead we relied on a control pull-down experiment to correct for false-positive prey-protein observations. Furthermore, we computed a pseudo-confidence score for the pull-down replicate data and validated the interaction data by: (i) assessing organization of the interactions into functional subnetworks; (ii) assessing the similarity of each bait profile with the control; (iii) comparing the gene co-expression of interacting and non-interacting pairs, and (iv) comparing orthologous interactions.

In total 130 distinct proteins were identified in the control JW801 strain in at least one replicate (Table S9). We used these data from the control pull-down experiments to account for the potential for non-specific interactions by the prey proteins identified in all bait pull-down experiments. 77 proteins identified in the control JW801 strain were used to adjust the bait pull-down data after summarizing the replicate pull-down data with a median-max statistic. We used this adjusted and normalized summary statistic for the emPAI protein abundance value as a pseudo-confidence score for observing protein interactions based on replicate LC-MS data (see Methods).

We were able to confirm that the emPAI scores performed reasonably on our data by assessing the emPAI values of the bait protein in each bait pull-down. It was expected that the tag-column specificity would result in enrichment of the tagged bait and its interacting partners and that these would have higher emPAI values than in the control fractions. In fact, the bait proteins were among the highest scoring proteins identified in the pull-down fractions (Table S2) with the exception of rubredoxin (Rub). Rub is a 52-amino acid protein containing no arginines and four lysines. Of these, three lysines are close to either the N- or C-terminus and the fourth lysine is followed by a proline residue, which can prevent cleavage by trypsin.

To assess statistical significance we calculated p-values for each pulled down protein by bootstrap analysis of the bait pull-down and control replicate data (Table S11). For the resampling we utilized all the available replicate data while excluding replicates used for the control if they were correlated with replicates of the bait in question (see Methods). 32 bait-prey interactions as well as 10 bait proteins were found to be significant (p<0.001, Table S11) and all 32 statistically significant interactions were present among the 134 interactions identified by the pseudo-confidence analysis. The 10 significant bait proteins further validate the results as bait proteins are expected to be the most abundant protein in a pull-down experiment. Our modified bootstrap analysis measures both how much greater the values were in the bait pull-down compared to the control as well as how specific a prey protein was for a given bait. The test is conservative in that we observed multiple bait proteins in the control data, thus some interactions were deemed not significant due to presence of bait-prey complexes in the control.

One approach to identifying potential false positives and negatives is to consider orthologous protein interactions in a related organism. We compared the *D. vulgaris* interactions for 6 of the 12 *D. vulgaris* baits that have orthologs in *E. coli*, to previously reported interactions in *E. coli*. In *E. coli*, these baits had 111 unique non self-self interactions, of which 89 involved prey that have orthologs in *D. vulgaris* and were pulled down by one of the six *E. coli* orthologs of the *D. vulgaris* baits. We identified 13 of these 89 (15%) “expected” interactions with high confidence (Fig. S2). In addition there were 31 high confidence protein interactions observed in *D. vulgaris* with orthologous bait and prey proteins in *E. coli* but for which no *E. coli* protein interactions were reported. Notably, no orthologous interactions were observed for NorV in *E. coli*, even though all of the interacting *D. vulgaris* proteins were assigned to *E. coli* orthologs. A number of DnaK and Pnp interactions were observed in *D. vulgaris*, which was not the case for the *E. coli* orthologs. It is not clear how conserved protein-protein interactions are between *E. coli* and *D. vulgaris*, as these species belong to different divisions of Proteobacteria. For example, Butland *et al.* found that only 14% of interacting pairs have a strong tendency to co-occur in other genomes. Below, we give an example of a complex that is not conserved in *E. coli* (the degradosome). Thus, recovering 15% of the ortholog-based expected interactions may be acceptable.

We performed an analysis of the confident interactions as well as the control data to assess similarity between the prey pull-down profiles for different bait proteins (Fig. S1). The prey protein pull-down profile for one of the baits (RoO) was highly correlated with the control (R = 0.71). Twelve prey proteins identified in the RoO pull-down data were also found in the control (no-bait pull-down) experiments, in addition to RoO itself. This suggests that RoO itself may have some interactions with the column explaining why the corresponding prey were also observed in the control. The other highest correlation coefficients corresponded to known complexes (ApsAB R = 0.91, RpoBC = 0.46) or a plausible interaction (RpoB-Pnp R = 0.55). For RpoB and Pnp it is also possible that the similarity in the expression profile is due to common binding partners to the nucleotide moiety of the native RpoB and Pnp proteins.

Considering all non-self interactions observed amongst the 12 bait proteins in this study, three reciprocal interactions were detected, giving a 50% (3 out of 6) confirmation rate for the interaction data by reciprocal bait pull-downs. The reciprocal interaction confirmation rates for *E. coli* were 8% (166 out of 2152) in the endogenous [10] and 0.06% (33 out of 5123) in the exogenous [9] pull-down experiments, although the numbers of baits in these experiments was much larger. A key difference in our study is that all of the reported interactions, including the reciprocal ones, were observed in triplicate.

**Figure 3:**
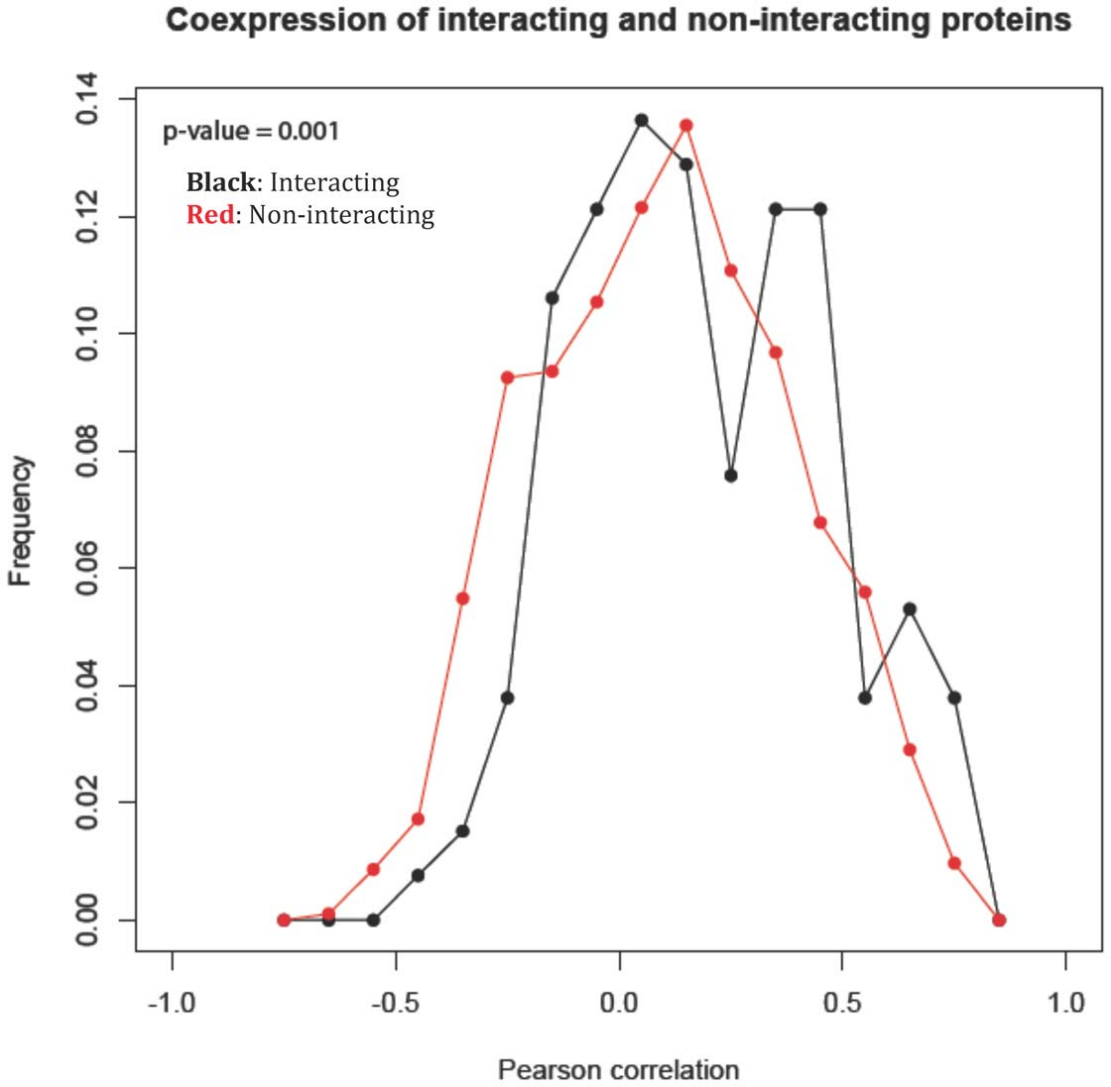
Gene expression correlations between interacting and non-interacting pairs in *D. vulgaris* Hildenborough. Shown are the *D. vulgaris* Hildenborough (JW801) gene co-expression distributions, measured as the centered Pearson correlation between vectors of gene expression values, for pairs of genes whose corresponding proteins were found to interact with high confidence (black) or not (red). The y-axis shows the fraction of all interacting or non-interacting protein pairs.

Interacting protein pairs would tend to be co-expressed as the presence of both proteins is necessary for formation of a complex, and vice versa for non-interacting pairs. The co-expression distribution (Fig. 3, Fig. S3 and Data S1) of the interacting pairs had a modestly higher mean than non-interacting ones (mean and standard deviation of 0.2±0.3 for interacting pairs vs. 0.1±0.3 for non-interacting pairs, two-tailed p-value = 0.001, (two sample t-test assuming unequal variance)). For co-expression (R≥∼0.3) there was an enrichment in interacting protein pairs, whereas for anti-co-expression and no co-expression (R≤∼0.2) there was an enrichment in non-interacting protein pairs.

### A network of protein-protein interactions

We reconstructed partial protein-protein interaction networks for both organisms. The interactions from *E. coli* K12 were restricted to only the baits and prey proteins observed in the JW801 dataset, which highlights the low orthology between the two species. The JW801 protein-protein interaction data consisted of 12 bait proteins having an average of 11.2 prey interactions per bait and a total of 90 prey proteins with an average of 1.5 bait interactions per prey, corresponding to a total of 134 interactions. The assignment of TIGR functional role membership to the protein interaction network revealed four functional subnetworks, all of which contained at least one bait protein interacting with other members of the functional role (Table S4). Over all interaction pairs, the TIGR functional role agreement was 23%. We used a permutation statistical test to determine significance of this arrangement of interactions into functional categories, given the functional role assignments of the proteins involved. We found that the observed agreement for the high confidence subset of interactions was higher than in the permuted data (16.0%, p-value = 0.017). For comparison, the functional role agreement in the interaction data including the prey proteins removed by the control pull-down data adjustment was 16% (13% in the permuted data, p-value = 0.04), i.e. identical to the permuted data for the highly confident subset of interactions. The largest of these subnetworks was ‘Central and intermediary metabolism’ consisting of eight proteins and including the two bait proteins ApsA and ApsB, which are known to interact with each other in *D. vulgaris* [24]. The ApsAB complex, or adenylylsulfate reductase, is absent in *E. coli* K12. The second largest functional subnetwork was ‘Energy metabolism’ with two baits CooH and RoO and a total of eight proteins. The ‘Transcription’ functional subnetwork consisted of five proteins including three baits: Pnp, RpoB, and RpoC. Of these RpoB and RpoC are expected to form a known complex in JW801. In the *E. coli* K12 ‘Transcription’ network, all of the detected interactions from JW801 are present and in addition the RpoC – RpoZ interaction was observed (Fig. S2). The ‘Protein fate’ subnetwork consisted of the bait DnaK along with three interactions.

A number of genes of unknown function had interactions with at least one of the functional subnetworks. To further investigate the putative functions of these 17 genes, we integrated various data sources to generate new hypotheses (Table S5). Interestingly, 7 of these proteins appear specific to the *Desulfovibrio* clade with no homologs above 50% sequence identity in other species and in some cases no homologs at all beyond close relatives of the *D. vulgaris* Hildenborough clade.

DVU0851, was pulled down by two baits, one of which, Rub, were proteins from the energy metabolism functional role. DVU0851 is the last gene in the *qmo* operon, which is supported by high gene expression correlations with all of the other five operon members [25]. DVU0851 appears to be evolutionarily recent with no homologs outside of *Desulfovibrio*, hence its function cannot be solely determined by the functional role of its operon since newly acquired genes often insert into operons with functionally unrelated genes [26]. Expression data confirm that DVU0851 is in the *qmo* operon, and the protein interaction data also suggest that it has some role in energy generation, even though it appears not to be associated with the Qmo complex.

DVU1455 was observed as an interacting partner of Pnp, along with several other nucleic acid binding proteins that co-eluted with Pnp. Examining the putatively assigned domain of this protein (COG1579) as well as assigned function of the neighboring protein (DVU1456: Transcriptional regulator) suggests a possible regulatory role for DVU1455.

Another intriguing observation was the co-elution of putative ATPase domain proteins (DVU2103 and DVU3330) with the heat shock protein DnaK. Finally, DVU2215 showed co-expression with other energy metabolism genes, suggesting that there are additional unknown features of energy generation in these anaerobic organisms that remain to be validated.

The network analysis and co-expression distribution discussed in the previous sections give us a broad view of the *D. vulgaris* interactome. In the following sections, we take a detailed look at individual baits and discuss the functional importance of associated interactions that were observed in this study. We discuss interactions associated with highly conserved proteins as well as those specific to *D. vulgaris*.

### Comparison of interactions for highly conserved proteins

We compared bait-prey interactions reported for *E. coli* K12 [10] (Table S3) by assigning the orthologous bait-prey protein interactions pairs from JW801. We chose tagged baits involved in essential cellular functions for which protein domains and their interactions would be expected to be conserved even in phylogenetically distant organisms. Three baits from the TIGR role of ‘Transcription’, and two from ‘Protein Fate’ were compared.

#### A. Strep-tagged RpoB (DVU2928) and RpoC (DVU2929) were found to be associated with known members of the highly conserved RNA polymerase complex (RNAP)

The tagged components of RNAP in *D. vulgaris*, RpoB and RpoC, are encoded by genes predicted to occur in an eight-member operon with genes encoding other proteins such as preprotein translocase (DVU2922), transcription antiterminator protein (nusG) and ribosomal proteins L11, L1, L10 and L7/L12 [27]. The gene for the α-subunit of RNAP (DVU1329) occurs in a separate operon that encodes several proteins of the small and large subunits of the ribosome. Using either *Strep*-tagged RpoB or *Strep*-tagged RpoC we observed all of the core catalytic components of RNAP - the α, β, β′ and ω subunits. However we did not observe other components such as sigma factors or accessory proteins perhaps due to the transient nature of those interactions.

In *E. coli* K12, proteins reported to co-purify with SPA-tagged RpoB (b3987) included the catalytic core composed of the α subunit (b3295) and the β′ (RpoC, b3988) subunit in addition to several sigma factors (σ^32^, σ^38^, σ^54^ and σ^70^), elongation factors (NusA, NusG), and accessory factors (RpoZ, HepA and YacL). Unlike RpoB (b3987) however, SPA-tagged RpoC (b3988) did not pull down sigma factors such as σ^32^, σ^38^, σ^54^ or YacL even though the core subunits were still observed, further suggesting the transient nature of the non-core component interactions in these complexes [10].

#### B. The chaperone machinery of D. vulgaris comprises DnaK (DVU0811), DnaJ (DVU1876, DVU3243), GrpE (DVU0812) and DafA (DVU1875)

In addition to serving as a molecular chaperone, the well-conserved protein DnaK also modulates heat-shock response in bacteria [1], [28], [29]. Proteins that co-eluted with *Strep-*tagged DnaK included HSPs from the ‘Protein fate’ role (TIGR) such as GrpE, two paralogs of DnaJ (DVU1876, DVU3243) and DafA (Table S12), all of which are predicted to be co-regulated during heat shock. The *D. vulgaris* Hildenborough genome indicates the presence of a third paralog of the gene for the molecular chaperone DnaJ (DVU1003), which did not co-purify with *Strep*-tagged DnaK. Of the three paralogs, only genes encoding the interacting partners DVU3243 and DVU1876 were over-expressed during heat shock response [1]. The relative transcriptional abundance ranking of *grpE*, *dnaJ* (DVU1876) and *dafA* suggest that these genes are not abundantly expressed in *D. vulgaris*, but their proteins were observed to associate with *Strep*-tagged DnaK (DVU0811).

In *E. coli* K12, heat shock proteins (HSPs) that co-purified with SPA-tagged DnaK (b0014) included GrpE (b2614), chaperone protein HscA (b2526), ATP-dependent protease Lon (b0439), and Peptidase B (b2523) but not the chaperones DnaJ (b0015) and GroEL (b4143) [10]. However, in other studies in *E. coli* K12, DnaK, GrpE, and DnaJ have been demonstrated to form a chaperone complex for *in vivo* repair of denatured proteins [28], [29], [30]. The *E. coli* K12 genome also features a second DnaJ homolog, CbpA (b1000), which can function as a co-chaperone and regulate the activity of the DnaK system. CbpA activity has been shown to be modulated by a small 11-kDa protein, CbpM (b0999). However, neither CbpA nor CbpM were identified in pull-down fractions of SPA-tagged DnaK even though DnaK itself was observed as prey for both SPA-tagged DnaJ and SPA-tagged CbpA [10]. In *Thermus thermophilus* the CbpM analog, DafA (TTHA1488), assembles the corresponding chaperones DnaK (TTHA1491) and DnaJ (TTHA1489) to produce a (DnaK)_3_–(DnaJ)_3_–(DafA)_3_ complex referred to as the KJA complex. DafA (TTHA1488), like its *E. coli* counterpart, inhibits the chaperone activities of both DnaK and DnaJ by forming the KJA complex and acts as a thermosensor under both heat stress and optimal growth conditions [31]. The resemblance of the DnaK (DVU0811)-DnaJ (DVU1876)-DafA (DVU1875) interaction to its *T. thermophilus* counterparts leads us to believe that a similar mechanism of DnaK regulation may be operative in this sulfate reducing bacterium (SRB).

The heat shock response in *E. coli* K12 is also characterized by up-regulation of a two-component ATP-dependent proteolytic complex comprised of adjacently encoded HSPs, HslV (b3932) and HslU (b3931) and the corresponding genes regulated by σ^32^ [32]. The respective homologs in *D. vulgaris*, HslV (DVU1577) and HslU (DVU1467), however, appear in separate predicted operons that lack σ^32^-dependent promoters or CIRCE sites that are present upstream of other heat-shock genes in this organism [33]. Our observations from this study also suggest that *Strep*-tagged HslV does not interact with HslU under the conditions we tested. This could be attributed to a weak association between the two proteins as reported previously [34]. In *E. coli* K12 however, using SPA-tagged baits, HslU and HslV have been identified in reciprocal tagging experiments [10] with the reported subunit composition of the protease complex being [(HslU)_6_]_2_[(HslV)_6_]_2_ [32], [35]. Even though upstream regions of *hslV* (DVU1577) and *hslU* (DVU1467) lack σ^32^ or CIRCE sites there is some evidence that these genes are co-regulated as both are highly over-expressed during heat shock and air stress [1], [4] and they are co-expressed overall with a correlation coefficient of 0.27, which is within the range of both interacting pairs (mean R = 0.2±0.3) and non-interacting ones (R = 0.1±0.3) in our study. We hypothesize that HslU (DVU1467) and HslV (DVU1577) may interact under stressor-specific conditions and function independently otherwise.

#### C. Strep-tagged, Polyribonucleotide nucleotidyltransferase (Pnp, DVU0503) interacts with ribosomal proteins but not with orthologs of components of the degradosome complex from E. coli K12

Polynucleotide phosphorylase (Pnp) is a 3′-to-5′ exonuclease and a 3′-terminal oligonucleotide polymerase. In *E. coli* K12, Pnp (b3164) is a component of the degradosome complex that plays an important role in messenger RNA processing and is composed of the following additional proteins: Ribonuclease E (Rne, b1084), RNA helicase (RhlB, b3780), polyphosphate kinase (Ppk, b2501) and enolase (Eno, b2779). The suggested component stoichiometries in the complex are [(Ppk)_4_][(Rne)_4_][(RhlB)_2_][(Pnp)_3_][(Eno)_2_] ([36]: www.ecocyc.org). While the assembled degradosome mediates the decay of transcripts in *E. coli* K12, the individual components have been suggested to be active in their unbound state as well [37]. Degradosome assembly in *E. coli* K12 is enabled by the C-terminal half of Rne, which provides a scaffold for other components of this protein complex, whereas the N-terminal half of Rne provides the catalytic function [38], [39]. The multiple sequence alignment of Rne from *D. vulgaris* (DVU3055) and its *E. coli* K12 counterpart (b1084) confirmed that only the N-terminal portion of Rne exhibits conservation (42% sequence identity) between the two species. Even though the *D. vulgaris* Hildenborough genome encodes several homologs to components of the *E. coli* K12 degradosome – Rne (DVU3055), RhlE (DVU1982) and Eno (DVU0322) – it is not entirely surprising that these potential interacting partners were not found complexed with *Strep*-tagged Pnp.

Proteins that co-purified with *Strep*-tagged Pnp included several members of the large and small subunits of the ribosome as well as DNA and RNA binding proteins. Similar interactions were also observed for SPA-tagged Pnp (b3164) in *E. coli* (Fig. S2) [10]. Direct or indirect interactions between Pnp (DVU0503) and ribosomal protein components could be interpreted to signal the existence of a larger complex of RNA-interacting proteins.

### Analysis of bait-prey interactions in D. vulgaris JW801

Bait-prey interactions for highly conserved proteins discussed in the previous section point to conserved interactions in most cases. Here we discuss the biological significance of the interactions associated with baits specific to *D. vulgaris* from the TIGR categories of ‘Central intermediary metabolism’ and ‘Energy metabolism’.

#### A. Methylation of sulfate reduction proteins and interactions with enzymes of the SAM cycle

Sulfate reduction in JW801 is carried out by the following cytoplasmic enzymes: ATP sulfurylase (Sat, DVU1295), inorganic pyrophosphatase (PpaC, DVU1636), the αβ heterodimeric adenylylsulfate reductase (ApsB, DVU0846 and ApsA, DVU0847), and the dissimilatory sulfite reductase composed of α, β, δ and γ subunits (DsrA, DVU0402; DvsB (a.k.a. DsrB in *D. vulgaris*), DVU0403; DsrD, DVU0404; and DsrC, DVU2776; respectively) [40]. Also known as desulfoviridin, the dissimilatory sulfite reductase complex from *D. vulgaris* has been reported to be an α_2_β_2_γ_2_ structure [41]. The *D. vulgaris* genome sequence reveals the presence of six possible membrane bound complexes involved in electron transfer – HmcABCDEF, TmcAB, OhcBAC, RnfABEDG, QmoABC and DsrMKJOP of which the last two are suspected to be involved in electron transfer to the sulfate reduction pathway [27]. Using *Strep*-tagged ApsA, we identified most of the cytoplasmically localized enzymes predicted to be involved in the sulfate reduction pathway described above (Table S12).

An interesting feature of several proteins in the sulfate reduction pathway was the presence of post-translational modifications (PTMs) in the form of methylated amino acids (Table 1). Protein methylation has been suggested to play a role in several biological functions such as protein-protein interactions, cellular localization, ribosome assembly, cell signaling and others [42], [43]. In this study, we identified mono-, di- and tri-methylated peptides from ApsB, ApsA, and DsrC. In addition, a conserved lysine residue from the ribosomal protein L7/L12 (DVU2927) was found to be methylated (Fig. S4), as reported in another study [44] for the *E. coli* K12 ortholog (b3986). The methylated lysine residues observed in this study appear to be very well conserved in close homologs of ApsB, ApsA, DsrC, and RplL, suggesting conservation of functionality (Fig. S5 & S6) for this post-translational modification.

**Table 1:** Post-Translational Modifications Identified in this study.

*Strep*-tagged	Interaction Partners	Peptide Sequence	Modification(s)	ProtScore	Percentile
Bait					Rank
DVU0846 (ApsB)	DVU0846 (ApsB)	SADSIMWTVK*FR	Trimethylation	2	99
	DVU0847 (ApsA)	FKDGYGPVGAWFLLFK*AK	Trimethylation	2	99
DVU0847 (ApsA)	DVU0847 (ApsA)	DGYGPVGAWFLLFK*AK	Trimethylation	2	99
		FKDGYGPVGAWFLLFK*AK	Trimethylation	2	99
		GPVGAWFLLFK*AK	Trimethylation	2	99
		PVGAWFLLFK*AK	Trimethylation	2	99
		FKDGYGPVGAWFLLFK*AK	Dimethylation	2	99
		DGYGPVGAWFLLFK*AK	Dimethylation	2	99
		DGYGPVGAWFLLFK*AK	Methylation	2	99
	DVU0846 (ApsB)	SADSIMWTVK*FR	Trimethylation	2	99
	DVU2776 (DsrC)	ESEGISDISPDHQK*IIDFLQDYYK	Trimethylation / Acetylation	2	99
		LK*EVYELFPSGPGK	Trimethylation+Oxidation	1.7	98
	DVU2927 (RplL)	TGLGLK*EAK	Methylation	2	99
		ALTGLGLK*EAK	Methylation	2	99
		IGVIK*VVR	Trimethylation	2	99
DVU2291 (CooH)	DVU2776 (DsrC)	LK*EVYELFPSGPGK	Trimethylation+Oxidation	1.7	98
DVU3185 (RoO)	DVU2776 (DsrC)	LK*EVYELFPSGPGK	Trimethylation+Oxidation	1.5	97
	DVU0847 (ApsA)	DGYGPVGAWFLLFK*AK	Trimethylation	2	99

Trimethylation has the same nominal mass shift as another PTM, acetylation. While the mass accuracy of our experiments was not sufficient to discriminate between the two PTMs in the MS^1^ scan, two pieces of evidence support these identifications as trimethylations. We observed mono- and di-methylation, +14 Da and +28 Da respectively, of the same peptide in fractions co-purified with *Strep*-tagged ApsA, suggesting that the +42 Da peptide is indeed tri-methylated and not acetylated. In addition, the MS/MS spectra revealed the presence of fragment ions corresponding to a neutral loss of 59 Da (Fig. S7), diagnostic for tri-methylation [45], [46].

Enzymes catalyzing these methylation reactions generally use S-adenosylmethionine (AdoMet) as the methyl (Me) donor, adding methyl groups to Lys or Arg [47]. Intriguingly, members of the *S*-adenosyl-L-methionine (SAM) cycle pathway (http://biocyc.org/META/NEW-IMAGE?type=NIL&object=PWY-5041) known to be implicated in methyl group transfers were observed as interacting partners of *Strep*-tagged ApsA. These included the S-adenosylmethionine synthetase (MetK, DVU2449) and adenosylhomocysteinase (AhcY, DVU0607) (Table S12).

Using *Strep*-tagged ApsB, we observed the larger subunit, ApsA, as an interacting partner, but none of the other enzymes involved in the sulfate reduction pathway or the SAM cycle components. However, differences in interacting members from reciprocally tagged baits are observed even for highly conserved complexes such as RNAP [10]. Site occlusion effects, conformation changes associated with the tag location, low protein abundance, and the detection limits of the mass spectrometry based approach employed in this study to exhaustively detect interaction partners, may be responsible for these apparent discrepancies. Nevertheless, the presence of SAM cycle components interacting with members of the sulfate reduction pathway suggests that methylation plays an important role in the energy metabolism of *D. vulgaris* although the exact biological implication of this finding remains to be elucidated.

#### B. The carbon monoxide-induced hydrogenase, CooH (DVU2291), and the carbon monoxide dehydrogenase, CooS (DVU2098), may play different metabolic roles in D. vulgaris from other bacteria

The genome sequence of *D. vulgaris* Hildenborough reveals the potential presence of two membrane-bound, cytoplasmically-oriented, hydrogenases, EchABCDEF and CooMKLXUH that could be involved in energy metabolism of this organism [27]. The transcriptional ranking of the *ech* genes (average relative expression rank of 34) is much lower than that for the *coo* genes (average relative expression rank of 90) during growth on LS4D (Table S10). We infer that, during lactate oxidation, CooMKLXUH may play a more prominent role in energy metabolism than the Ech complex in this SRB.

**Figure 4:**
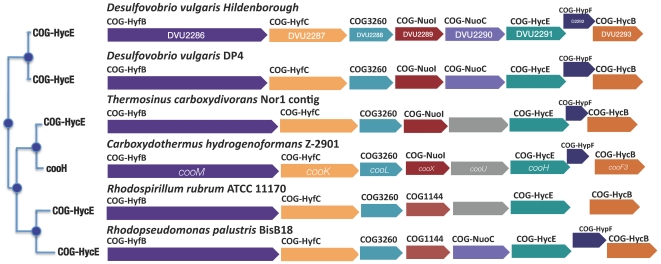
Conservation of the operon encoding DVU2291 between the δ-proteobacteria, the α-proteobacteria and the Clostridia.

We tagged the cytoplasmically localized hydrogenase from the Coo complex to identify interacting partners of this protein. CooH is predicted to be located in an eight-gene operon regulated by a CO-sensing activator, CooA [33]. The tree and genome browsers on www.microbesonline.org reported proteins with conserved COG assignments and synteny information for this predicted operon in δ-Proteobacteria (*D. vulgaris* Hildenborough and *D. vulgaris* DP4), α-Proteobacteria (*Rhodopseudomonas palustris* BisB18 and *Rhodospirillum rubrum* ATCC11170), and Clostridia (*Carboxydothermus hydrogenoformans* Z-2901). Thus, these data indicate a horizontal gene transfer event among these clades (Fig. 4). In *R. rubrum* COG3261 (carbon-monoxide dehydrogenase, catalytic subunit) and COG3640 (carbon monoxide dehydrogenase accessory protein) are key enzymes involved in conversion of carbon monoxide to carbon dioxide and hydrogen when carbon monoxide is used as the sole energy source [48]. Among the sequenced δ-Proteobacteria, only *Desulfovibrio* species have *coo* operons or the CooA regulator (DVU2097).

In *D. vulgaris*, COG3261 and COG3640 are located in an operon separate from that containing CooH and it is also apparently regulated by CooA [33]. In *C. hydrogenoformans*, the CO-oxidizing:H_2_-evolving enzyme complex activated by CO was shown to be composed of seven subunits – two catalytic sites, a CO-oxidizing site and a H_2_-forming site (COG1151), which are connected via different iron–sulfur cluster containing electron transfer subunits (COG3261, COG852, COG1142, COG1143 and COG3260) [49]. The corresponding genes in *D. vulgaris* (CooS (DVU2098), CooH (DVU2291), CooU (DVU2290), CooF (DVU2293), CooX (DVU2289), and CooL (DVU2288)) might be expected to form a similar complex. However, under the conditions we tested for protein complexes, only the CooU subunit of the hydrogenase from this putative complex was pulled down by CooH. The membrane-bound components of this complex may not have been observed also due to the nature of the extraction protocol used for bait purification. In contrast, several members of the energy metabolism network were observed to interact with CooH notably desulfoviridin, the αβ adenylylsulfate reductase, and their interacting partners (Fig. 2).

*cooS* does not appear to be abundantly transcribed during normal growth (percentile rank: 55, Table S10). Also there was no appreciable expression correlation (R = 0.03, MicrobesOnline release 28) between the two *coo* operons harboring CooS and CooH respectively ([25]: MicrobesOnline). The lack of strong transcript co-expression in addition to the lack of interaction between the corresponding proteins suggests that CooH and CooS have different functions in *D. vulgaris* during growth on LS4D medium. While CooH appears to be a constitutive hydrogenase involved in hydrogen cycling during growth on LS4D medium, it remains to be seen whether the presence of CO affects transcript expression levels of CooS such that the two proteins could interact.

#### C. Interacting partners of Rubredoxin, Rub (DVU3184), and Rubredoxin-oxygen oxidoreductase, Roo (DVU3185), differ widely from those of Pyridine nucleotide-disulfide oxidoreductase, Nox (DVU3212)

In this study we tagged three oxidoreductases from *D. vulgaris* that have been suggested to be involved in the oxygen defense mechanism of this anaerobe. Rubredoxin oxygen oxidoreductase (Roo), rubredoxin (Rub), and desulfoferrodoxin (Sor/Rbo, DVU3183) are part of one such oxidative stress defense system. Recent work on Roo and Sor/Rbo knock-out mutants suggests an important role of the former under microaerobic conditions and the latter under aerobic conditions [50]. Among chromosomally-encoded proteins reported to be involved in oxygen defense [51] relative transcript abundance of *sor*, *rub* and *roo* under anaerobic conditions are among the highest (percentile rank >95%).

Based on operon organization it has been suggested that Sor/Rbo and Roo may collaborate in the reduction and the detoxification of oxygen entering the cytoplasm through the use of Rub as a common electron donor [51]. We observed Sor/Rbo to co-purify with tagged bait Roo lending support to the aforementioned hypothesis (Table S12). Interestingly, Sor/Rbo was also observed to interact with other baits in this study including ApsA, Ftn, and CooH. Several members of the energy metabolism network including desulfoviridin, the αβ adenylylsulfate reductase and QmoAB copurified with Roo.

NADH oxidase (Nox – COG446) acts on NADH and transfers electrons to an acceptor and has been suggested to contribute to antioxidant activities in anaerobes. Biochemical characterization of purified Nox (DVU3212) suggests that its flavin mononucleotide (FMN) cofactor reduces oxygen to hydrogen peroxide and transfers electrons to adenylylphosphosulfate (APS) reductase from NADH [52]. Based on this result, Nox has been suggested to play a role in both oxygen defense and sulfate reduction [52]. Consistent with the former role, an apparent homolog to Nox from *D. desulfuricans* B-1388 has been shown to be induced under low oxygen partial pressures [53]. Consistent with the latter role, close homologs of Nox are found adjacent to the dissimilatory sulfite reductase, DsrA (COG2221), in distantly related bacteria (e.g., *Desulfitoacterium hafniense*, *Clostridium difficile*). In JW801, however, under normal growth conditions we did not find tagged Nox to interact with energy metabolism proteins to a significant degree. Unlike Sor/Rbo, Nox appears to be isolated from the energy metabolism network of this SRB (Fig. 2, Table S2). Based on the current evidence, we infer that oxygen defense may be the primary function of Nox. The different interacting partners between the oxidoreductases Nox and Roo point to the variety of electron transfer routes in this model sulfate reducer.

For organisms with no neighboring species for which protein-protein interaction data have been collected, the accepted ‘gold standard’ comparison approach to assess data quality is problematic. If in addition the dataset in question is relatively small, there is no accepted way to estimate the number of false positives and false negatives. We present a method designed to directly assign confidence to protein-protein interactions based on experimental data from pull-down experiments. A series of functional genomic and comparative analysis confirm that the high confidence subset of our data is of high quality, including a high reciprocal interaction index compared to previous studies, a significant co-expression of the interacting proteins, and a higher enrichment for functional role interactions compared to random. Our protein-protein interaction data from this study highlight several metabolic features that appear unique to *D. vulgaris*. Highly conserved proteins between *D. vulgaris* and *E. coli* K12, such as RpoB, RpoC, and DnaK, display several conserved interacting partners. In contrast, structural differences between the ribonuclease, Rne, from *D. vulgaris* and *E. coli* K12 may explain why only a subset of interactions are conserved for polynucleotide phosphorylase, Pnp, even though the corresponding bacterial genomes encode for most partners of the degradosome complex. The interaction network contrasts the vastly different energy generation schemes of JW801 with *E. coli* K12 and this difference clearly contributes to the absence of many orthologs in the latter. Interestingly, proteins from the sulfate reduction pathway (ApsA, ApsB, and DsrC) are found to be methylated, which may be attributed to SAM cycle components observed to co-purify with these proteins. The methylated lysine residues from these proteins are highly conserved in other bacterial species suggesting a potentially conserved functionality of this modification. In the absence of added carbon monoxide and during growth on LS4D, CooH is a constitutively expressed hydrogenase and does not appear to interact with CooS. This result is in opposition to observations made for the corresponding orthologs from *R. rubrum* and *C. hydrogenoformans*. The oxidoreductases Sor and Rub are characterized by their high constitutive expression levels as compared to other chromosomally encoded proteins implicated in oxygen reduction and ROS detoxification, and interact with many other redox enzymes.

The single-crossover approach we describe in the current study is restricted to monocistronic operons or genes located relatively close to the terminal ends of their respective operons. The complete chromosomal integration of the plasmid bearing the tagged gene as currently configured can cause polar effects on promoter-distal genes. A non-integrative double crossover approach is being perfected to tag any gene on the chromosome regardless of its operon location that will contribute to a complete protein network of this model organism. While our approach represents progress towards the confident identification of protein-protein interactions by setting a rigorous standard for experimental design, data collection and data analysis, a number of obstacles remain. Protein interactions *in vivo* span a wide range of binding affinities and they can be finely regulated in a condition-dependent manner. Thus to obtain high coverage of the protein interactome, it will be necessary to analyze multiple growth conditions and to devise ways to uniformly collect data both for weak, transient interactions as well as constitutive complexes. In addition a complete view of the interactome will require distinguishing between protein isoforms and post-translational modifications. Finally, the affinity purification followed by MS method has a key limitation, namely the inability to directly distinguish direct physical interactions from secondary interactions, e.g. interactions through other proteins. Further work on experimental design and computational methods is necessary to address these shortcomings.

## Materials and Methods

### Strains and media

Strains used in this study are listed in Table S1. All % concentrations are wt/vol unless otherwise indicated. *Escherichia coli* (TOP10 or α-select) strains were cultured in SOC medium (components per liter of medium: 5 g yeast extract, 9 g tryptone, 0.5 g sodium chloride, 0.19 g potassium chloride, 3.6 g glucose, 10 ml of 1 M magnesium chloride, and 10 ml of 1 M magnesium sulfate) or LC medium (components per liter of medium: 10 g tryptone, 5 g sodium chloride, and 5 g yeast extract) at 37°C. For solid media, 15 g agar were added per liter. To select for kanamycin-resistant *E. coli* cells, kanamycin was added to LC medium to a final concentration of 50 µg/ml. Chemicals and antibiotics were obtained from Fisher Scientific (Pittsburg, PA). Plasmids bearing the tagged targets were constructed using established cloning techniques and then electroporated into competent JW801 cells followed by selection for G418 resistance (described below). A 202-Kb native plasmid, pDV1, containing 157 predicted ORFs, is found in wild type *D. vulgaris* Hildenborough and has been lost, generating strain JW801. The aforementioned genes do not affect the ability of strain JW801 to grow on LS4 medium containing 0.1% yeast extract or LS4D medium, which is completely defined [3]. However, JW801 displays higher transformation efficiency than wild-type *D. vulgaris* Hildenborough for *E. coli* K12-derived plasmids. This may be due to the loss of a type II restriction endonuclease (DVUA0020) predicted to be encoded in pDV1; hence this host was chosen for this study.

Following electroporation, JW801 constructs were allowed to recover at 30°C in an anaerobic growth chamber (Coy Laboratory Products, Grass Lake, MI) in LS3 medium, which is LS4 modified by elimination of sulfate and addition of 40 mM Na_2_SO_3_ as the terminal electron acceptor. To identify putative JW801 affinity-tagged constructs, cells were plated into molten sulfate-containing medium, LS4D [3], containing 1.5% agar. During selection and culturing transformants, G418 (RPI corp., Mt. Prospect, IL) was added to a final concentration of 400 µg/ml. G418 was used in place of kanamycin because it was more effective for selection of the kanamycin resistance marker in JW801.

### Plasmid construction

For construction of *Strep*-tagged (IBA, St. Louis, MO) *D. vulgaris* genes and their introduction into the sulfate-reducer, pKASK was constructed by digestion of the pASK-IBA3 plasmid (IBA, St. Louis, MO) with *Mfe*I (New England Biolabs, Ipswich, MA) for insertion of a kanamycin resistance cassette. The neomycin-kanamycin resistance gene, *neo*, located on the 1.8-Kb EcoRI fragment from pUC4-KIXX (Amersham Biosciences, Piscataway, NJ) was gel purified with the QIAEXII Gel Extraction kit (Qiagen, Valencia, CA) and ligated with the *Mfe*I-digested pASK-IBA3 generating pKASK.

The pKASK vector or pCR4Blunt-TOPO (Invitrogen, Carlsbad, CA) was used to introduce tagged genes into the chromosome of JW801. Three different cloning schemes were used to generate the plasmids introduced by electroporation, as described below. Tagging plasmids were sequenced to verify that the correct fragment was amplified and that no errors were introduced during the cloning procedure. All sequencing was performed at the University of Missouri DNA core facilities (http://www.biotech.missouri.edu/dnacore/). The sequences returned were aligned with the published *D. vulgaris* Hildenborough genome sequence (http://www.ncbi.nlm.nih.gov/entrez/viewer.fcgi?db=nucleotide&val=AE017285.1).

In *scheme one* (Fig. S9), primers were designed to amplify the desired gene with specific restriction enzyme sites included on each end (Tables S7, S8). The PCR reaction was performed with *Pfu* polymerase (Stratagene, La Jolla, CA), the amplicon was captured in a plasmid (pGEM T-Easy, Promega, Madison, WI; or pCR4Blunt-TOPO), and the resulting plasmid transformed into prepared *E. coli* K12 cells (α-select, Bioline, Randolph, MA; or TOP10, Invitrogen, Carlsbad, CA) (Table S8). The plasmid with the inserted PCR product was isolated, the amplicon digested, and the correct amplicon fragment isolated by separation on a 0.8% agarose gel for gel- purification. The DNA fragment containing the gene of interest was ligated in-frame into an appropriately digested pKASK, transformed into chemically competent *E. coli* K12 cells (α-select), and purified from kanamycin resistant transformants.

In *scheme two* (Fig. S9), primers were designed to amplify a target gene with the 5′ end of the reverse primer containing the complementary sequence of the *Strep*-tag. PCR was performed with *Pfu* polymerase, the amplicon captured in the pCR4Blunt-TOPO vector, and the resulting plasmid transformed into *E. coli* TOP10 cells. Kanamycin resistant colonies were grown and the corresponding plasmids were isolated. Location of the forward primers for the first two schemes varied depending on the location of the gene within an operon (Table S8). If a gene was monocistronic or the last gene in an operon, the forward primers began at the start codon of the gene. If a gene was the first in an operon or located in the middle of an operon, the forward primer was designed to amplify approximately 300 bp upstream of the putative start codon of the first gene in the operon to obtain promoter sequences and to ensure wild-type expression of the genes downstream in the operon.

*Scheme three* (Fig. S9) was utilized only for tagging of *cooH* (DVU2291) and *rub* (DVU3184). This scheme was developed to tag a gene in the middle of an operon (when scheme two was not permissible) and was designed to allow wild-type expression of the downstream genes. In order to place the tag on the 3′ end of a complete copy of the gene, a three PCR approach was used. PCR-1 amplified a region of DNA upstream of the gene that we presumed should contain the promoter. PCR-2 amplified the gene with the in-frame C-terminal tag. Finally, PCR-3 generated a composite of the first two PCR products using Splicing by Overlap Extension (SOEing; [54]) that introduced the *Strep*-tag onto the 3′ end of a complete copy of the gene controlled under its native promoter. This scheme was designed so that in JW801 a recombination event in either the upstream region or within the gene itself would introduce a complete version of the tagged gene into the chromosome.

### Transformation of JW801 strains

Electroporation of the plasmids into JW801 was performed as previously described [55] (see Methods S1).

### Selection and storage of JW801 strains expressing affinity-tagged proteins

In the anaerobic chamber, well separated colonies expressing the antibiotic resistance of the introduced vector were transferred into 0.5 ml of Wall LS3 medium [14] containing the selective antibiotic, grown overnight, diluted into 5 ml of the same medium, and again grown overnight. From this culture, 1.5 ml of cells was collected for the preparation of genomic DNA. Three freezer stocks were made from the remaining 3.5 ml by addition of glycerol to a final concentration of 10% (vol/vol). Samples of 0.75 ml were transferred into cryogen vials that were stored at −80°C.

### Southern blots

In order to verify that plasmid integration occurred at the predicted location, a Southern blot was performed. Genomic DNA was prepared using the Wizard Genomic DNA Purification Kit (Promega, Madison, WI) from 1.5 ml of culture grown anaerobically to early stationary phase in Wall LS3 medium. DNA was quantified with a ND-1000 spectrophotometer (Nanodrop, Wilmington, DE). Genomic DNA (2–5 µg) from wild-type cells and those with putatively tagged genes were digested at 37°C for 3 h with 5–10 units of a restriction enzyme (New England Biolabs, Ipswich, MA or Promega, Madison, WI) (Table S7). Restriction enzymes were chosen such that a single band would be visualized for the wild-type control DNA and two bands would be visualized for the DNA of the correctly integrated tagged construct when probed with the target gene. Separation of digested DNA, transfer onto Zeta-probe membrane (Bio-Rad, Hercules, CA), and Southern probing were performed as previously described [56] (see Methods S1). Band size was determined by comparison to the distance of its migration to those of the DNA fragments in the 1-Kb DNA ladder standard (NEB) as visualized on an agarose gel.

### Growth of JW801 strains and soluble protein extraction

Three one-liter cultures of each JW801 strain producing tagged proteins (Table S6) were grown anaerobically in LS4D medium containing G418 at 400 µg/ml [3]. Cells were harvested at late log phase (final optical densities are listed in Table S8) as described previously [1] (see Methods S1) and the resulting pellets were washed once with 100 mM Tris-HCl, pH 8.5 and stored at −80°C until analyzed. Prior to lysis, frozen cell pellets were suspended in Buffer W (100 mM Tris-HCl, 150 mM NaCl, 1 mM EDTA, pH 8) containing a protease inhibitor cocktail consisting of Na_2_EDTA (0.5 mM), pepstatin (10 µM), bestatin (0.13 mM), and Pefabloc SC plus (Roche Applied Science, Indianapolis, IN) (0.4 mM). Soluble protein extractions were prepared from these cells by sonication as described previously [1] (see Methods S1). Protein samples were maintained below 4°C at all times. Total protein concentrations (Table S8) were determined using the bicinchoninic acid protein assay (Pierce, Rockford, IL) using bovine serum albumin as the standard.

### Enrichment of Strep-tag® fusion proteins

Protein complex purifications were performed using a 1-ml *Strep*-Tactin® Sepharose column (IBA, St. Louis, MO) as per the manufacturer's recommendations and briefly outlined here. All steps were carried under gravity flow at 4°C. After the *Strep*-tactin® column was equilibrated, cell lysates (10 ml) containing the protease inhibitor cocktail were added to the column. Total protein mass loaded on to the *Strep*-tactin® column was between 60∼70 mg. After the cell extract had completely entered the column, the loaded column was washed 5 times with 1 ml of Buffer W to remove unbound proteins. Tagged targets and associated proteins were eluted from the column using 3 ml of buffer containing desthiobiotin, which competes with the binding of *Strep*-tag II to *Strep*-Tactin, the engineered streptavidin. Six 500-µl fractions were collected and stored at −80°C until further use.

### Protein sample analysis

To determine the presence of the affinity-tagged target and any associated proteins, eluted protein fractions were subjected to MS analysis after in-solution tryptic digestion as follows. To 50 µl of the eluted fractions, 2 µl of 100 mM DTT was added. The tubes were heated to 95°C for 15 min and then placed on ice for 10∼15 min. Five µl of Trypsin Gold (100 ng/µl) (Promega, Madison, WI) was added to each sample and the mixture incubated at 37°C overnight. Digested peptides were then analyzed by reversed-phase LC-MS/MS on an Eksigent nanoLC-2D system (Eksigent, Dublin, CA) coupled to a Quadrupole-Time Of Flight (Q-TOF) mass spectrometer (QSTAR ELITE Hybrid Quadrupole TOF, Applied Biosystems, Framingham, MA) described previously [57]. On the QSTAR ELITE system, 3 µl of the digested proteins earlier eluted from *Strep*-Tactin were injected onto a PepMap100 trapping column from a Famos Autosampler (Dionex-LC Packings, Sunnyvale, CA). Peptide separation took place on a Dionex PepMap 100 column (75 µm×15 cm) at a flow rate of 300 nl/min. Following a 7 min wash period with buffer A (2% (v/v) acetonitrile, 0.1% (v/v) formic acid), the sample was eluted with a gradient, 5 to 35% buffer B (98% (v/v) acetonitrile, 0.1% (v/v) formic acid) in 30 min, followed by 35 to 80% (v/v) buffer B in 10 min, and then 80% (v/v) buffer B for 10 min. The column was re-equilibrated by a decreasing gradient of buffer B, 100 to 5% (v/v), in 5 min, that was maintained for 20 min.

The LC system was interfaced to the QSTAR mass analyzer via a nanospray source equipped with a 15 µm Picotip emitter (New Objective, Woburn, MA) operating in the positive ion mode (2300–2400 V). Data were collected with Analyst™ QS 2.0 (Applied Biosystems, Framingham, MA) and Information Dependent Acquisition (IDA; Applied Biosystems, Framingham, MA). The three most abundant multiply-charged ions from a 0.25-s MS survey scan (350–1600 amu) above a threshold of 50 counts were selected for IDA analysis. Selected ions were isolated in Q1 (resolution = LOW) and were fragmented with rolling collision energy. MS/MS scans were collected over a mass range of 100–1600 amu set with a fragment intensity multiplier of 4.0 and maximum accumulation time of 2.5 sec. Parent ions (within 100 ppm) and isotopes were excluded from subsequent IDA selection for 60 s following one repeat analysis. The mass spectrometer was tuned and calibrated from the product ion spectrum with [Glu^1^] fibrinopeptide D [M+2H]^2+^ prior to analysis.

For several of the pull-down samples we verified the proteins identified by one peptide in the LC-MS (Q-Star) analysis by a multiple-reaction-monitoring (MRM) LC-MS experiment on an Applied Biosystems 4000Q-Trap mass spectrometer using similar column conditions. Since we did not have protein standards from which to optimize MRM transitions, a list of MRM transitions was generated by the MIDAS program (Applied Biosystems, Framingham, MA) for each protein. The MRM transitions were limited to *m/z* range 400–1200, 2+ and 3+ charge states. The resolution of both Q1 and Q3 were set to “unit”, and each transition was measured for 50-ms dwell time per cycle. No more than 100 transitions were used for each LC run to limit the total MRM cycle time to five seconds. An IDA method, triggered above 500 counts/s, was used to verify the peptide identity via collision-induced dissociation (CID) for each MRM transition.

### Mass spectrometer (MS) data analysis

Mascot Distiller (v 2.1) was used to sum similar precursor ion scans from each LC-MS/MS run and generate product ion peak lists for subsequent database searches. A Mascot MS/MS Ion Search (Mascot v 2.1, MatrixScience, London, UK) was performed for each dataset against a protein database consisting of all putative ORF sequences of *D. vulgaris* Hildenborough (JW801) ([25]: MicrobesOnline release as of 02/08/05, 3503 predicted protein-coding genes, see additional Data S2) appended with trypsin, bovine serum albumin, and common contaminants. Only fully digested peptides with up to one missed cleavage site were considered. Oxidation of methionine was considered as a variable modification. Precursor and product ion tolerances were set at ±100 ppm and ±0.2 Da, respectively. Results were extracted to Excel and filtered to retain only top ranked peptide matches with a match expectation value of p≤0.05 for each query. The list was further filtered to retain only sequences from the highest scoring protein in the few cases where multiple sequence matches passed the first filter for a given spectrum. Protein abundance in each sample was estimated from the Exponentially Modified Protein Abundance Index (emPAI) [18] values obtained from Mascot.

Post-translational modifications (PTMs) were identified by searching the data with ProteinPilot 2.0 (Applied Biosystems, Framingham, MA). ProteinPilot was chosen for the PTM search over Mascot because the Paragon search algorithm [58] searches for modifications based on probabilities without having to specify the search space in advance. Consequently, the breadth of PTMs considered was much greater. Each dataset was searched using the same protein database as was used for the protein identification (see above) with the following settings: protease digestion with trypsin, cysteine blocking with iodoacetic acid (as appropriate to the sample), confidence level was set to 95% (ProtScore = 1.3), and the Paragon algorithm was set to “thorough” with biological modifications considered. The MS/MS spectra for all reported PTMs were manually evaluated for accuracy.

### Network reconstruction

The total emPAI data for all bait pull-down experiments were collected into a matrix, where the columns were bait pull-down experiment fraction replicates and the rows prey proteins (Table S12). Each prey protein was found in at least one bait pull-down-experiment fraction replicate, indicating that it was present in the cell pellet lysate and thus was available to be pulled down by other baits. Since we relied on triplicate observations and experimental control data we also considered proteins for which only one peptide was observed. JW801 by itself lacks the *Strep*-tag II sequence and thus serves as a control for proteins pulled down by any tagged bait protein. In order to assess the false positives introduced in the protein-protein interaction data by proteins with potential for non-specific interactions, we tested a *Strep*-Tactin column with cell lysate from JW801 and identified proteins eluting from the column from three fractions that were collected in the same way as the tagged-bait pull-down fractions. Pseudo-confidence scores for each interaction pair or protein observed in the no-bait pull-down control were computed by first taking the maximum observed emPAI value in any elution fraction for a given bait pull-down biological replicate. Taking the maximum value accounts for the fact that the same elution fraction in different replicates may represent different parts of the elution profile. In order to account for proteins interacting non-specifically with the column, this maximum emPAI value was adjusted by subtracting the median of the maximum values for the corresponding protein in the no-bait control experiment. Next, the median of these adjusted maximum emPAI-values was computed across the three biological replicates; we call these the median-max emPAI values. Finally, the adjusted median-max values for all prey proteins in each bait pull-down were divided by the median-max value observed for the bait protein or the maximum value of any prey in the bait pull-down if the bait was not observed. We call this normalized adjusted median-max emPAI value the pseudo-confidence score for observing a prey protein in a bait pull-down. The prey protein pull-down profile is a vector of pseudo-confidence scores across the series of bait pull-down experiments.

The high confidence subset of protein interactions included only proteins observed in all three biological replicate experiments for each bait pull-down. To produce conservative estimates, we used all of the median-max emPAI values of the protein observations from the no-bait control regardless of how many no-bait control replicates the proteins were observed. In all of the no-bait control samples, RoO (DVU3185) was observed with the highest overall emPAI value. On comparing the median-max data for prey proteins associated with tagged RoO to the data for proteins present in the control we observed that 85% were in common. Proteins, which were present in all three replicates of the control and at equal or higher emPAI values in the tagged RoO pull-down data, were considered to be interacting with RoO. The same rules were applied to ApsA (DVU0847), which was also observed in the control and with the second highest emPAI value of bait proteins from this study. Tagged ApsA had a 63% overlap of prey proteins with the control. Thus each high confidence prey protein was observed in all three biological replicates for at least one bait pull-down and with a non-zero median-max emPAI value greater than or equal to the median-max emPAI value observed for that protein in the control.

The *D. vulgaris* Hildenborough protein-protein interaction network was visualized using Pajek [59] and subsequent vector graphics editing (Fig. 2). Each edge corresponds to an observed interaction between a bait and a prey protein and the width of the edge corresponds to the pseudo-confidence score. For the cases of prey proteins observed with same median-max emPAI value in the bait pull-down experiment as in the no-bait pull-down experiment, the edge is represented as a dashed line and the edge width is arbitrary (0.001). Nodes in the network were colored by TIGR functional categories and edges were colored if the two nodes connected by the edge shared a TIGR role. TIGR categories were assigned as described previously [25]. The TIGR classification is incomplete and does not include a number of characterized protein families. There can also be incompleteness and ambiguities in TIGR function assignments as TIGRFAM protein families are biased towards aerobic bacteria.

### Interaction data analysis

We computed replicate pull-down experiment agreement as the fraction of prey in common between pairs of pull-downs. We report the agreement for the total emPAI dataset and for the dataset after control subtraction (Fig. S8). For the total data, the agreement ranged from 35% (DVU3212) to 69% (DVU2928) and for the data after control subtraction 0 (control) to 72% (DVU2928 bait). The agreement increased in 4 cases after control subtraction (DVU3212, DVU0846, DVU2929, and DVU2928) but for most baits control subtraction led to a decrease in agreement, with a mean decrease of 14%. This decrease in agreement is explained by the fact that often the proteins in common between those associated with tagged baits and the control, i.e. mostly those with nonspecific interaction potential, showed high replicability.

To study similarity between the bait protein pull-down fractions a Pearson correlation coefficient was calculated for each pair of bait proteins, treating the pseudo-confidence scores (or median-max emPAI values for the control data) of proteins observed in the pull-downs as vectors of corresponding values. This bait-bait prey profile correlation analysis heatmap was rendered with JColorGrid [60].

A reciprocal pair interaction is defined as an interaction between a pair of proteins A and B where both of the proteins were used as a bait and each bait pulls down the corresponding interacting partner, that is A pulls down B (A-B) and B pulls down A (B-A). A reciprocal interaction confirmation rate was computed by dividing the number of reciprocal bait-prey interactions that were observed by the number of reciprocal interactions that were possible to be observed in the dataset (the latter corresponding to “viable” bait and prey proteins [61]). We define possible reciprocal interactions to be ones for which at least one half of the reciprocal interaction was observed, e.g. for a reciprocal interaction between proteins A and B, protein A must pull down protein B and/or protein B must pull down protein A. To assess statistical significance for each observed interaction, we performed a bootstrap analysis [62] by resampling the replicate data maximum fraction emPAI values. As ‘control’ data for each bait we used all other bait and control pull-down replicate data which where correlated with R<0.3 with any of the pull-down replicates for that bait. The resampling was done 10,000 times, each time sampling with replacement three values from the bait replicate data and three values from the ‘control’ data. We counted the number of times *n* that the median of the bait values was higher than the median of the ‘control’ values and reported the p-value as 1−*n*/10,000 (Table S11).

To assess the biological significance of the interaction network we used a measure of functional role agreement consisting of the number of interacting pairs sharing a functional role divided by the total numbers of interacting pairs. The p-value for observing the arrangement of interactions in functional categories was obtained by permuting the TIGR functional role assignments for each protein and recomputing the functional agreement. This was repeated 100,000 times, and the reported p-value is the number of times the functional role agreement in the permuted data was greater than the observed functional role agreement.

### Sequence ortholog assignments

The orthologous *E. coli* K12 interactions were based on the previously published pull-down data [10], [13] using the set of *E. coli* K12 orthologs for the *D. vulgaris* Hildenborough bait and prey proteins found in this study. Orthologs were determined by reciprocal best BLAST matches, where the matches from both organisms had an e-value< = 0.0001. Blastpgp version 2.2.9 was used for BLAST searches with default parameters except z = 100000000. A number of *D. vulgaris* Hildenborough genes did not have *E. coli* orthologs, and these were omitted from the *E. coli* K12 network (Fig. S2). Coverage of orthologous *E. coli* interactions by the *D. vulgaris* interactions was computed as the number of *D. vulgaris* interactions with an orthologous interacting pair in *E. coli* divided by the total number of *E. coli* interacting protein pairs with *D. vulgaris* orthologs which were a bait or prey protein in this study.

### Transcriptomic analyses

Transcript abundance was computed as the mean Log2 ratio of mRNA to gDNA hybridization intensities, normalized as described previously [3]. Only untreated conditions were used and the list of experiments included in this calculation can be found elsewhere [63]. Mean Log_2_ ratio values were converted to percentile rank, with the highest percentile corresponding to maximum observed expression. We define the average relative expression rank of a gene as the percentile rank of the mean log_2_ ratio value.

For the gene-gene co-expression analysis, *D. vulgaris* Hildenborough expression data from 106 experimental comparisons were used (time point series from different culture treatment and control comparisons (see [63] for list of conditions). Co-expression was calculated as the centered Pearson correlation between normalized (as reported previously, [1]) expression profiles of two genes. The co-expression values for interacting and non-interacting pairs (see Data S1) were plotted using a frequency polygon (Fig. 3). The Welch two-tailed t-test p-value assuming unequal variances was used to assign statistical significance to the difference between the interacting and non-interacting protein-gene co-expression distributions.

## Supporting Information

Figure S1 Pairwise correlations of bait proteins based on pulled-down prey protein profiles. Pearson correlation coefficients were computed for all pairs of bait proteins as well as the no-bait pull-down control based on the pulled-down prey protein pseudo-confidence profiles (median-max for control). Positive correlations indicate that the bait proteins have similar protein pull-down profiles.
(PDF)

Figure S2 Interactions in *E. coli* K12 [11], [14] and *D. vulgaris* between proteins that have *D. vulgaris* orthologs in *E. coli* and which involve the *D. vulgaris* bait proteins used in this study. The edge thickness corresponds to protein interaction confidence in *E. coli* (arbitrarily 50 if only observed in Butland *et al.*, otherwise confidence from Hu *et al.*). Black dotted edges indicate interactions observed in *E. coli* but not in *D. vulgaris*. Thick black solid edges indicate interactions observed in *E. coli* and in *D. vulgaris*. Thin solid edges indicate interactions detected in *D. vulgaris* but not *E. coli*.
(PDF)

Figure S3 Gene co-expression correlations between interacting pairs in *D. vulgaris* Hildenborough. Shown are the gene co-expression Pearson correlations for the confident protein interactions identified in this study (Fig. 2), The thickness of the edges corresponds to the confidence value for the interaction and the color of the edges corresponds to the gene expression correlation value.
(PDF)

Figure S4 CLUSTAL 2.0.8 multiple sequence alignment of Ribosomal protein L7/L12. Note: Boxed region shows conserved lysine that has been observed to be methylated in RplL from *E. coli* K12 [44] (Arnold and Reilly, 2002) as well as *D. vulgaris* JW801.
(PDF)

Figure S5 Multiple Sequence Alignment of ApsA (DVU0847), ApsB (DVU0846), DsrC (DVU2776) and RplL (DVU2927).
(PDF)

Figure S6 Hidden Markov Model Alignments. Note: Boxed region indicates position of methylated lysine residue observed in this study.
(PDF)

Figure S7 Q-Star MS/MS data for ApsA, ApsB, and DsrC post-translational modifications.
(PDF)

Figure S8 Replicate agreement for pull-down experiments.
(PDF)

Figure S9 Cloning schemes used for suicide vector construction.
(PDF)

Table S1 *Strep*-tagged baits chromosomally integrated in JW801.
(XLS)

Table S2 Bait-Prey Interactions in JW801.
(XLS)

Table S3 Bait-Prey Interactions in *E. coli* K12 corresponding to orthologous baits from JW801.
(XLS)

Table S4 Functional protein-protein interaction subnetworks in JW801.
(XLS)

Table S5 Evidence for hypothetical protein functional associations.
(XLS)

Table S6 Strains and plasmids used in this study.
(XLS)

Table S7 Primers used for PCR amplification, Southern probe, and sequencing.
(XLS)

Table S8 Primer set, plasmid(s), restriction enzyme(s), and *E. coli* strain used to make each construct.
(XLS)

Table S9 Proteins identified from control sample of *D. vulgaris* Hildenborough (JW801).
(XLS)

Table S10 Relative transcriptional abundance ranking.
(XLS)

Table S11 Bootstrap Analysis.
(XLS)

Table S12 emPAI values for all pull-down experiments in this study.
(XLS)

Data S1 Gene expression correlations for interacting pairs in this study.
(TXT)

Data S2 Protein sequence data for *D. vulgaris*.
(FASTA)

Methods S1 Supplementary Methods.
(DOCX)
