# Supplementary material for: Towards a Rigorous Network of Protein-Protein Interactions of the Model Sulfate Reducer Desulfovibrio vulgaris Hildenborough
Source: PLoS One. 2011 Jun 28;6(6):e21470. doi: 10.1371/journal.pone.0021470 (PMC3125180; doi:10.1371/journal.pone.0021470)
Supplement: Figure S1 — Pairwise correlations of bait proteins based on pulled-down prey protein profiles. Pearson correlation coefficients were computed for all pairs of bait proteins as well as the no-bait pull-down control based on the pulled-down prey protein pseudo-confidence profiles (median-max for control). Positive correlations indicate that the bait proteins have similar protein pull-down profiles. (PDF) [file pone.0021470.s001.pdf]

Figure S1: Pairwise correlations of bait proteins based on pulled-down protein profiles.

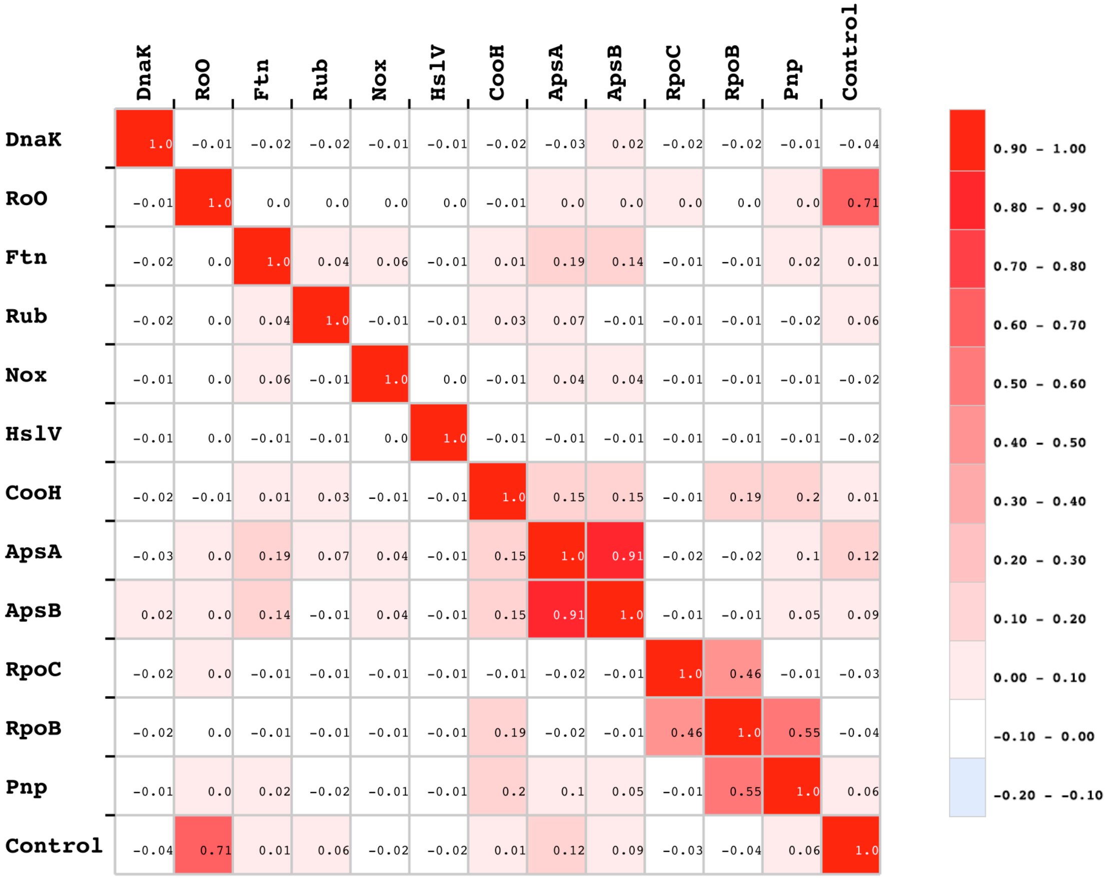

Pearson correlation coefficients were computed for all pairs of bait proteins as well as the control fraction based on the pulled-down protein profiles. Positive correlations indicate that the bait proteins have similar pulled-down protein profiles. ApsA (DVU0847) – ApsB (DVU0846), RpoC (DVU2928) – RpoB (DVU2929) and RpoB (DVU2929) – Pnp (DVU0503) are all highly correlated with each other. The control fraction correlations provide information on baits with potential for false positives and in this study the maximum correlations between the baits and control are small (RoO (DVU3185) being the exception (see Methods)) after removing the prey identified as having false positive potential.
