# Supplementary material for: Towards a Rigorous Network of Protein-Protein Interactions of the Model Sulfate Reducer Desulfovibrio vulgaris Hildenborough
Source: PLoS One. 2011 Jun 28;6(6):e21470. doi: 10.1371/journal.pone.0021470 (PMC3125180; doi:10.1371/journal.pone.0021470)

Figure S3: Gene expression correlations between interacting and non-interacting pairs in *D. vulgaris* Hildenborough.

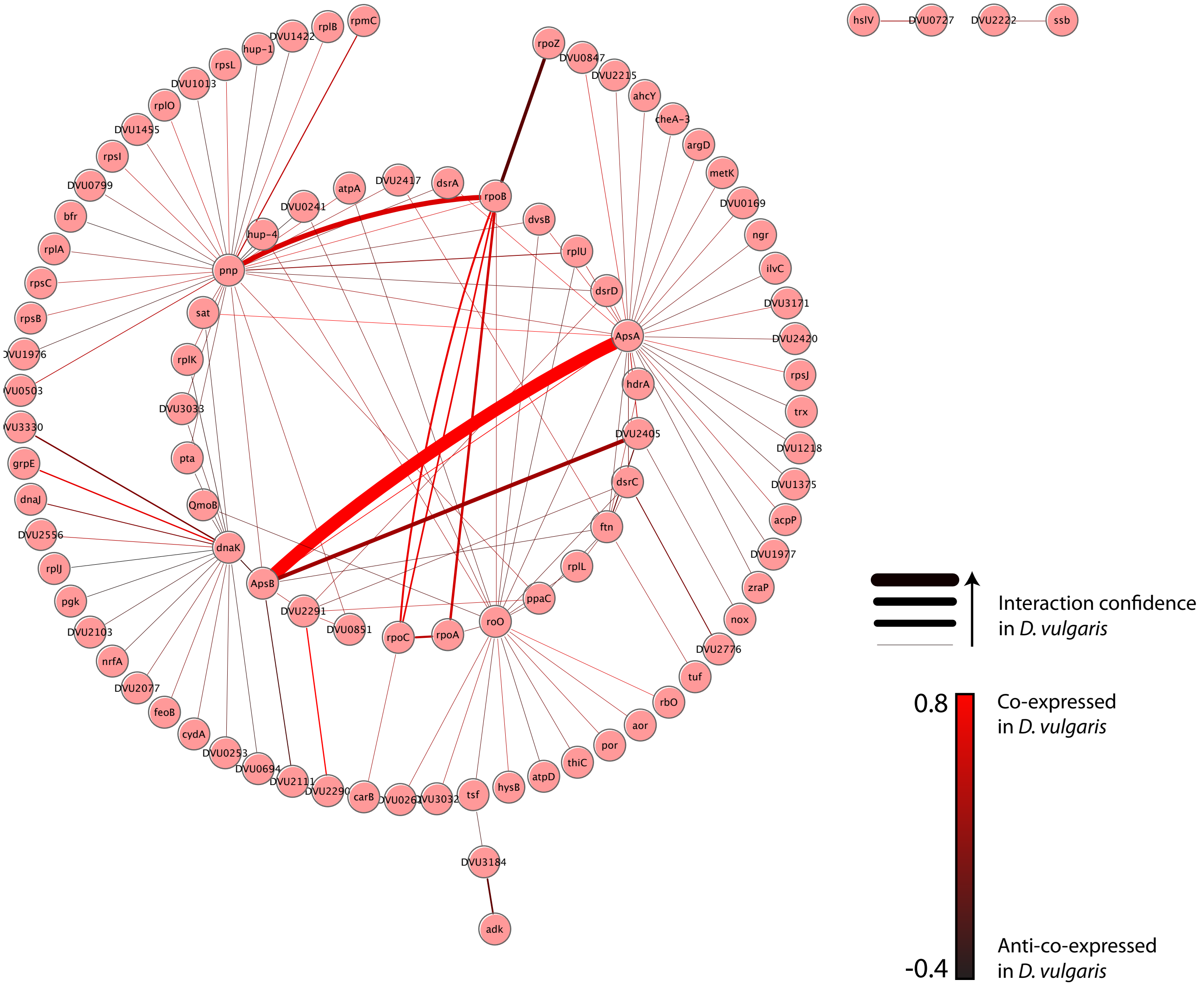

Supplement: Figure S3 — Gene co-expression correlations between interacting pairs in D. vulgaris Hildenborough. Shown are the gene co-expression Pearson correlations for the confident protein interactions identified in this study (Fig. 2), The thickness of the edges corresponds to the confidence value for the interaction and the color of the edges corresponds to the gene expression correlation value. (PDF) [file pone.0021470.s003.pdf]
