# Supplementary material for: Towards a Rigorous Network of Protein-Protein Interactions of the Model Sulfate Reducer Desulfovibrio vulgaris Hildenborough
Source: PLoS One. 2011 Jun 28;6(6):e21470. doi: 10.1371/journal.pone.0021470 (PMC3125180; doi:10.1371/journal.pone.0021470)
Supplement: Figure S4 — CLUSTAL 2.0.8 multiple sequence alignment of Ribosomal protein L7/L12. Note: Boxed region shows conserved lysine that has been observed to be methylated in RplL from E. coli K12 [44] (Arnold and Reilly, 2002) as well as D. vulgaris JW801. (PDF) [file pone.0021470.s004.pdf]

Figure S4. CLUSTAL 2.0.8 multiple sequence alignment of Ribosomal protein L7/L12

```
RplL_Dv         MSITKEQVVEFIGNMTVLELSEFIKELEEKFGVSAAAPMAAMAVAAPAGDAAPAEEEKTE 60
RplL_Ec         MSITKDQIIEAVAAMSVMDVVELISAMEEKFGVSAAA-----AVAVAAG-PVEAAEEKTE 54
                *****:*::* :. *:*::: *:*. :**********     ***..** .. * *****

RplL_Dv         FDIILKSAGANKIGVIKVVRALTGLGLKEAKDKVDGAPSTLKEAASKEEAEEAKKQLVEA 120
RplL_Ec         FDVILKAAGANKVAVIKAVRGATGLGLKEAKDLVESAPAALKEGVSKDDAEALKKALEEA 114
                **:***:*****:.***.**. ********** *:.**::***..**::**  ** * **

RplL_Dv         GAEVEIK 127
RplL_Ec         GAEVEVK 121
                *****:*
```

Note: Boxed region shows conserved lysine that has been observed to be methylated in RplL from *E. coli* K12 (Arnold and Reilly, 2002) as well as *D. vulgaris* JW801.
