# Supplementary material for: Towards a Rigorous Network of Protein-Protein Interactions of the Model Sulfate Reducer Desulfovibrio vulgaris Hildenborough
Source: PLoS One. 2011 Jun 28;6(6):e21470. doi: 10.1371/journal.pone.0021470 (PMC3125180; doi:10.1371/journal.pone.0021470)
Supplement: Figure S5 — Multiple Sequence Alignment of ApsA (DVU0847), ApsB (DVU0846), DsrC (DVU2776) and RplL (DVU2927). (PDF) [file pone.0021470.s005.pdf]

Figure S5: Multiple Sequence Alignment of ApsA (DVU0847), ApsB (DVU0846), DsrC (DVU2776) and RplL (DVU2927):

S. FIG 5A: CLUSTAL 2.0.8 multiple sequence alignment: ApsA (DVU0847)

```
DvDP4           MPMIPVKEQPKG--VAIAEPTVKEHDVDLLIVGGGMGACGTAFEAVRWADKY----APEL 54
DvH             MPMIPVKEQPKG--VAIAEPTVKEHDVDLLIVGGGMGACGTAFEAVRWADKY----APEL 54
DdG20           --MIPVKEQVKG--VAIAEPTIKEHDVDILLVGGGMGACGTAFEAVRWADKY----APEL 52
DpLSv54         -LNMALPNKPKGEILAVVDPEIVEHDIDVLIVGGGMAACGTAFEIKKWAP-------ADL 52
Td              ---------MAG---EFGNPEVVQEDVDVLLIGGGMACCGAGYEIMRWADAAKAELGMDL 48
CcCaD3          ------MG-EKNEFKYCKKPEVVHVDTDILLIGGGMACCGAAYEAAKWATPK------GL 47
PpBU-1          ------MGVEKNEFKFCKKPEVVYIDTDILLIGGGMACCGAVYEAAKWATPK------GL 48
CtTLS           ------MGVEKNEFKFSQKPEVVYVDTDILLIGGGMACCGAAYEAAKWATPK------GL 48
DaMP104C        ----------------MPNFETVEVTTDLLIVGGGMAACGAAVEAAHWAKKH------GL 38
DrMI-1          ----------------MPNFETVVVNTDILILGGGMAACGAAVEAAYWAKKQ------GL 38
SfMPOB          ----------------MPNFETVVVETDLLIIGGGMSACGAAFEAAYWAKKH------GL 38
                                  .        *:*::****..**:  *   **          *

DvDP4           KILLIDKASLERSGAVAQGLSAINTYLG----KNDADDYVRMVRTDLMGLVREDLIFDLG 110
DvH             KILLIDKASLERSGAVAQGLSAINTYLG----KNDADDYVRMVRTDLMGLVREDLIFDLG 110
DdG20           KILLIDKAALERSGAVAQGLSAINTYLG----DNSADDYVRMVRTDLMGLVREDLIFDLG 108
DpLSv54         KIMLVDKAALERSGAVAQGLSAINTYIG----ENKIEDYVKMVRNDLMGVVREDLIYDCG 108
Td              KIKLVDKAAMDRSGAVAQGLSAINTYIGS---EQDPADYARMVSNDLMGITRDDLAYDLG 105
CcCaD3          KITMVDKASVDRSGAVAQGLSAINTYCG----ENDPADYVKYVRTDLMGIIREDLVYDLG 103
PpBU-1          RITMVDKASVDRSGAVAQGLSAINTYCG----ENDPADYVKYVRTDLMGIIREDLVYDLG 104
CtTLS           RITMVDKAAVDRSGAVAMGLSAINTYCG----ENDPADYVKYVRADLMGIIREDLVYDLG 104
DaMP104C        KVTLVDKAAMDRSGAVAQGLSAINLYVGLRDNENTVMDYVEYVRNDLMGIARDDLVYNIA 98
DrMI-1          KITLVDKAAMDRSGAVAMGLSAINQYLGIAKGENTVEDYVKYVKNDLMGIARDDQVADIA 98
SfMPOB          KVTVVDKAAMDRSGAVAMGLSAINQYIGYAKGENTLEDYVKYVRQDLMGISREDLVYNIA 98
                :: ::***:::****** ****** * *    .:   **.. *  ****: *:*   : .

DvDP4           RHVDDSVHLFEEWGLPCWIKDEHGHNLDGAQAKAAGKSLRNGDDPVRSGRWQIMINGESY 170
DvH             RHVDDSVHLFEEWGLPCWIKDEHGHNLDGAQAKAAGKSLRNGDDPVRSGRWQIMINGESY 170
DdG20           RHVDDSVHLFEEWGLPCWIKDENGHNLDGAAAKAAGKSLRNGDAPVRSGRWQMMINGESY 168
DpLSv54         RHVDESVKLFEEWGLPVWKKDAAGDNLDGSKPAAS---LREGGTPVRTGKWQIMINGESY 165
Td              RNVDDSVHLFEEWGLPIWKTDENGERHDGAQGLPA---LKDGGKPVRSGKWQIMINGESY 162
```

```
CcCaD3          RHVDDSVHLFEEWGLPVWKRDEDGSSMDGSKPAPK---LTEGGKPVRSGRWQIMINGESY 160
PpBU-1          RHVDNSVHLFEEWGLPVWKRDEDGSTQDGAKPAPK---LSEGGKPVRSGRWQIMINGESY 161
CtTLS           RHVDNSVHLFEEWGLPIWKRDEDGSTMDGAKPAPK---LTEGGKPVRSGRWQIMINGESY 161
DaMP104C        RHVDSTVHLFEKWGLPIWPKGEDDPFRI--------------GPYQRGGRWQVMINGESY 144
DrMI-1          RHVDSSVHLFEKWGLPIWTNDE--------------------GNYVRGGRWQIMLNGESY 138
SfMPOB          RHVDSSVHLFEKWGLPIWKDAD--------------------GKYVHEGRWQIMINGESY 138
                *:**.:*:***:**** *                        .   : *:**:*:*****

DvDP4           KCIVAEAAKNALGEAR--------IMERIFIVKLLLDANTPNRVAGAVGFNLRANEVHIF 222
DvH             KCIVAEAAKNALGEAR--------IMERIFIVKLLLDANTPNRVAGAVGFNLRANEVHIF 222
DdG20           KNIVAEAAKNALGEDR--------IMERIFIVKLLLDANEENRIAGAVGFSLRENVVHIF 220
DpLSv54         KPIVAEPAATALGADN--------ILERVFIVKLLLDKNKPNQIAGAAGFSTRDNTVHIF 217
Td              KWIVAEATKKALGMDR--------IEERIFIVKLVNDKNDPSRIAGAVGFSTRDHKVVVY 214
CcCaD3          KVIVAEAAKKALEYNRKATGVDQNLFERVFISELIFDKNDPSKVAGAIGFSVREHKAYVF 220
PpBU-1          KVIVAEAAKKALEFNRKATGVDQNLFERVFISELIHDKNDPNRIAGAIGFSVRENKAYVF 221
CtTLS           KVIVAEAAKKALEYNRKETGVEQNLYERVFISELIHDKNNPSKVAGAIGFSVREHKAYVF 221
DaMP104C        KIIVAEAAKNALG--------KDNIYERVFIVEPIVENN---RIAGAVGFSVREEKFYVF 193
DrMI-1          KIIVAEAAKNALG--------TDNIYERVYICEPIMDGD---RIAGAIGFSTREEKVYVF 187
SfMPOB          KVLVAEAAKNAMATLGD----KGELLERVFIVEPLMDGD---KCVGGVGFSVRENKFYVI 191
                * :***.: .*:            : **::* : : : :   : .*. **. * .   :

DvDP4           RSNAMLVACGGAVNVYKPRSTGEGMGRAWYPVWNAGSTYTMCAQVGAEMTMMENRFVPAR 282
DvH             RSNAMLVACGGAVNVYKPRSTGEGMGRAWYPVWNAGSTYTMCAQVGAEMTMMENRFVPAR 282
DdG20           RTNAMLVACGGAVNVYKPRSTGEGMGRAWYPVWNAGSTYTMCAQVGAEMTMMENRFVPAR 280
DpLSv54         RCKAAMVACGGAVNIFRPRSTGEGKGRAWYPVWNAGSTYTMCAQVGATLTMIENRFTPAR 277
Td              KAKAILLAAGGCVNIFRPRSVGEGTGRAWYPVWNAGSTYAMAAEAGAELTMMENRFVPAR 274
CcCaD3          TAKTMLLACGGAVNIYRPRSTAEGQGRAWYPIWNAGTTYALAAQAGCELVLMENRFVPAR 280
PpBU-1          TAKTMLLACGGAVNVYRPRSTAEGQGRAWYPVWNAGTTYALAAQAGCELVLMENRFVPAR 281
CtTLS           TAKTMLLACGGAVNVYRPRSTAEGQGRAWYPVWNAGTTYALAAQAGCELVLMENRFVPAR 281
DaMP104C        RAKAVLCAMGGCVGVFRPRSSGEGLGRSWYPPFSTGSSAYFTIKAGAEMTCQEVRFIPVR 253
DrMI-1          KAKAVLASMGGAVGVFKPRSTGEGLGRSWYPPFSSGSSAYFTMVAGAEMTCQEVRFIPVR 247
SfMPOB          KAKATIAAMGGAVHVFRPRSVGEGLGRAWYPPWNAGSTAYFTIRAGAEMTCQEVRFIPVR 251
                  :: : : **.* :::*** .** **:*** :.:*::  :   .*. :.  * ** *.*
```

```
DvDP4           FKDGYGPVGAWFLLFKAKATNYKGEDYCATNRAMLKPYEDRGYAKGHVIPTCLRNHMMLR 342
DvH             FKDGYGPVGAWFLLFKAKATNYKGEDYCATNRAMLKPYEDRGYAKGHVIPTCLRNHMMLR 342
DdG20           FKDGYGPVGAWFLLFKAKATNFRGEDYCVTNRAMLKPYEDRGYAKGHVIPTCLRNHMMLR 340
DpLSv54         FKDGYGPVGAWFLLFKAKVANGLGEYYAMGDSAKEELAQFMPYGASPVTPTCLRNHLMIK 337
Td              FKDGYGPVGAWFLLFKAQAVNAYGEVYMQKNKELLNDYP--PYGQAAVPASCLRNHLMLK 332
CcCaD3          FKDGYGPVGAWFLFFKCKATNSLGEDYCATNLATANKDFGKYAEDPHKLTTAMRNHMMMI 340
PpBU-1          FKDGYGPVGAWFLFFKCKATNSLGEDYCVTNLATANKDFGKYAEDPHKLTTAMRNHMMMI 341
CtTLS           FKDGYGPVGAWFLFFKCKATNSLGEDYCATNLAAANKDFGKYAEDPHKLTTAMRNHMMMI 341
DaMP104C        FKDAYGPVGAWFLLFKSVATNAFGENYMVTNAAELEKWG--KYGKVKPIPANLRNYLGMI 311
DrMI-1          FKDAYGPVGAWFLLFKSRAVNGFGEEYMVTRRPELDNWG--PYGKVKPIPANLRNYLGML 305
SfMPOB          FKDGYGPVGAWFLLFKSRAVNAFGEEYMVTRANELPKWA--PYGLAKPIPANLRNWLGME 309
                ***.*********:**. ..*  ** *                      .: :** : :

DvDP4           EMREGRGPIYMDTKTALQTTFANMTPEQQ---------KHLESEAWEDFLDMCVGQANLW 393
DvH             EMREGRGPIYMDTKTALQSTFANMTPEQQ---------KHLESEAWEDFLDMCVGQANLW 393
DdG20           EMREGRGPIYMDTKTALQTTFETMTPAQQ---------KHLESEAWEDFLDMCVGQANLW 391
DpLSv54         ELKEGRGPIYMATDVALNAFLDERKEAGLDDKSLKKFWKHLESEAWEDFLDMSVGQAGLW 397
Td              EMQEGRGPIYMDTVTALAKLRETLTPREV---------KHLEAEAWEDFLDMCVGQCGIW 383
CcCaD3          DMKAGKGPILMRTHEAMAALGESMTPKQM---------KHLEAEAWEDFLDMCIGQAVVW 391
PpBU-1          DMKAGKGPILMRTHEAMQALGETMTPKQM---------KHLEAEAWEDFLDMCIGQAVVW 392
CtTLS           DMKAGKGPILMRTHEAMAALAETMTPKQI---------KHLEAEAWEDFLDMCIGQAVVW 392
DaMP104C        DVSAGKGPIYMQTADAIKRLAAKYEGDEK---AFKKKMKHLHDEAWEDFLDMTISQALLW 368
DrMI-1          DVSDGKGPLFMETDEAIANLAKKYEDDPK---AFKKKMKELENEAWEDFLDMTVAQAILW 362
SfMPOB          DVMAGKGPIYMKTEEAIANLAAKYKDDPK---AFKKKMKELESEAWEDFLDMTISQALLW 366
                ::  *:**: * *  *:                     *.*. ********* :.*. :*

DvDP4           ASMNIQPEERGSEIMPTEPYLLGSHSGCCGIWVSGPDEKWVP------EDYKVRASNGK- 446
DvH             ASMNIQPEERGSEIMPTEPYLLGSHSGCCGIWVSGPDEKWVP------EDYKVRASNGK- 446
DdG20           ACMNIQPEEVGSEIMPTEPYLLGSHSGCCGIWTSGPDEEWVP------EDYKVRAKNGK- 444
DpLSv54         AGMNIEPEKVGSEIMPTEPYMLGSHSGCCGIWTSGPDEAWVP------EVANESRSHKYK 451
Td              VGENIEPEKKNSELMPTEPYLLGSHSGCCGIWVSGPEDVGAPTDEAHADAAKIPAHLPKG 443
CcCaD3          AGNNIEPDKSPSELMPTEPYLLGSHAGCAGIWVSGPGD-----------ISGIPSEWSWG 440
PpBU-1          AGNNIEPDKTPSELMPTEPYLLGSHAGCAGIWVSGPDD-----------LKGFPAEWNWG 441
CtTLS           AGNNIEPEKTPSELMPTEPYLLGSHAGCAGIWVSGPGD-----------IAGVPAEWSWG 441
```

```
DaMP104C        AACNINPEEKESEIAAAEPYFIGSHSGASGAWVSGPDD-----------VAPPEY--FWG 415
DrMI-1          AASNIAPEEKHSEIAAAEPYFIGSHSGASGAWVSGPAD-----------IAPAEY--QWG 409
SfMPOB          AGSNIQPEEKPSEIAAAEPYFIGSHSGASGAWVCGPED-----------VMPAEYKKQWE 415
                .  ** *::  **: .:***::***:*..* *..** :

DvDP4           ---IYNRMTTVEGLWTCADGVGASGHKFSSGSHAEGRICGKQMVRWCLDHKDYKPAIKES 503
DvH             ---IYNRMTTVEGLWTCADGVGASGHKFSSGSHAEGRICGKQMVRWCLDHKDYKPAIKES 503
DdG20           ---VYNRMTTVEGLWTCADGVGASGHKFSSGSHAEGRICGKQMVRWCIDHKDYKPAIAEK 501
DpLSv54         W--GYNRMTTVDGLFTAGDGVGASGHKFSSGSHAEGRIAAKQMVKFCRDN-DFTPELMQT 508
Td              WNWGYRSMTTVKGLFTAGDGVGASGHKFSSGSHAEGRMCAKSMVKYCIDNKDWKPELDTP 503
CcCaD3          ----YNRMTTVEGLFTAGDGVGASGHKFSSGSHAEGRIAGKAMTAYCLDHADYKPTLGRD 496
PpBU-1          ----YNRMTTVDGLFTAGDGVGASGHKFSSGSHAEGRIAGKNMTAYCLDHVDYKPELGRD 497
CtTLS           ----YNRMTTVDGLFTAGDGVGASGHKFSSGSHAEGRIAGKSMTAYCLDHADYKPELGRD 497
DaMP104C        ----YPNLTTVDGLFAAGDASGASSHKFSSGSHAEGRIAGKAACKYLVDKNPDLPNVDPA 471
DrMI-1          ----YTNMTTVKGLFAAGDASGASSHKFSSGSHAEGRIAGKSMVRFVVENN-QEPNIDDA 464
SfMPOB          PIGVYANMTTVKALFTCGDGSGASSHKFSSGSHAEGRVAAKGAIAYILDNN-VAPKVDDA 474
                    *  :***..*::..*. ***.************:..*    :  ::    * :

DvDP4           -ADELVKLIYRPYYNYMEGKAASTDPVVNPSYITPKNFMMRLVKCTDEYGGGVGTYYTTS 562
DvH             -ADELVKLIYRPYYNYMEGKAASTDPVVNPSYITPKNFMMRLVKCTDEYGGGVGTYYTTS 562
DdG20           -GEDLAKLIYRPYYNYLAGKDASTDPVVNPEYITPKNFMMRLVKCTDEYGGGVGTYYTTS 560
DpLSv54         -PQEIADEIYAPVRLYNEHVGASTAADVNPNYCKPAGLMMRLMKATDEYGGGVATYYMTS 567
Td              -VEQLVEEIYKPVRTFLEHKDYTTAIDVNPNYITPKMLQFRLQKIMDEYVAGVATYYKTN 562
CcCaD3          -VDEVLGEIYAPMETFGKYKDYSTDPNVNPNYIRPKMFQARLQKIMDEYVAGVATWYTTS 555
PpBU-1          -VEEVIAEIYAPMEIFGKNKDYSTDPNVNPNYIRPKMFQARLQKIMDEYVAGVATWYTTS 556
CtTLS           -VDEVIAEIYAPMETFAKYKDYSTDPSVNPNYIRPKMFQARLQKIMDEYVAGVSTWYTTS 556
DaMP104C        VVEALKAKILAPLDRFQEFKDFTTAPEVNPNYILPKQYKHRIQKLMDEYAGGWGSNFTTS 531
DrMI-1          KVEELKAKILKPLAVWEEFAGFTTDPETNPNYILPKMYMFRLQKMMDEYAGGVVANFTTS 524
SfMPOB          KVEALKKIILAPMDRFEEFAKLSSDPDINPNFIKPKMFMFRLQKIMDEYCAGVSSQFTTN 534
                  : :   *  *   :      ::    **.:  *     *: *  *** .*  : : *.

DvDP4           AAALDTGFSLLGMLEEDSLKLAARDLHELLRCWENYHRLWTVRLHMQHIRFREESRYPGF 622
DvH             AAALDTGFSLLGMLEEDSLKLAARDLHELLRCWENYHRLWTVRLHMQHIRFREESRYPGF 622
DdG20           QAALDTGFHLLDMLEEDSLKLAARDLHELLRCWENYHRLWTVRLHMQHIAFREESRYPGF 620
DpLSv54         GKLLNICLDLLLLLREDADKMAAGDLHELLRCWENYHRIWCVETHIRHIEFRKESRYPGF 627
Td              ANMLAVAEDKLGMLKEDAEKMRAKDLHELLRAWENYHRILTAEAHMKHIQFREESRYPGF 622
CcCaD3          KTMLDKGLEHLSLLKEDAEKMAAEDVHELMRCWENYHRVIAGEAHARHILFREDSRYPGY 615
PpBU-1          KTMLEKGLEHLSLLKEDASKMAAEDNHELMRCWENYHRLMAGEAHARHILFREDSRYPGY 616
```

```
CtTLS           KTMLEKGLEHLSLLKEDAEKMAAADLHELMRAWENYHRLMAGEAHARHILFREDSRYPGY 616
DaMP104C        KMLLEKGMEYLQMLKEDSEKLAARDLNELMRCWENIHRTWVAESHIRTILFREETRWPGY 591
DrMI-1          KTLLEVGLELLTMLKEDSQKLAARDLHELMRAWENIHRTLQAESHIRTVLFREETRFPGY 584
SfMPOB          KSLLTRGLELLTMLKEDSEKLAASDLHELLRAWENTHRLWIAESHLRHVMFREESRWPGY 594
                   *      * :*.**: *: * * :**:*.*** **    . * : : **:::*:**:

DvDP4           YYRADFMGLDDSKWKCFVNSKYDPATGETKIFKKAYYQIIPE--------- 664
DvH             YYRADFMGLDDSKWKCFVNSKYDPATGETKIFKKAYYQIIPE--------- 664
DdG20           YYRADFMGLDDSKWKCFVNSRYDVKAGTTKVFKKPYYQIVPA--------- 662
DpLSv54         YYRSDFPNVDDENWKCFVNSTFNPETKEWLCEKVECLNIIETEPWL----- 673
Td              YYRMDKNFVDEENWHCFVNSVYDKNSKQWNCFKRAHVDLVDKSKLFKPAAH 673
CcCaD3          YFRADHFYVDDENWKCFTISKYDRESKNWTLSKRDYVQIIPD--------- 657
PpBU-1          YFRADHFYVDDENWKCFTISKYDRSTQNWTLSKRDYVQVIPD--------- 658
CtTLS           YFRADHFYVDDENWKCFTISKYDRDSKEWTLSKRDYVQVVPD--------- 658
DaMP104C        YFRADFPNMDDN-WEVFVNCKWDPNTGEWEVFKRDIIRL------------ 629
DrMI-1          YFRADLPKMDPD-WEVFCNCKYDAANGEWEMIKRPIIRLVD---------- 624
SfMPOB          YYRADFPDMDEANWKCFVNSVYNPATGEWTMKKVPVVNIV----------- 634
                *:* *   :*   *. *  . ::         *     :
```

Note: Boxed region indicates position of lysine residue that was found to be methylated in ApsA (DVU0847). Homologs from the following organisms were used for this alignment: *Desulfovibrio vulgaris* DP4 (DvDP4), *Desulfovibrio vulgaris* Hildenborough (DvH), *Desulfovibrio desulfuricans* G20 (DdG20), *Desulfotalea psychrophila* LSv54 (DpLSv54), *Thiobacillus denitrificans* (Td), *Chlorobium chlorochromatii* CaD3 (CcCaD3), *Pelodictyon phaeoclathratiforme* BU-1 (PpBU-1), *Chlorobium tepidum* TLS (CtTLS), *Desulforudis audaxviator* MP104C (DaMP104C), *Desulfotomaculum reducens* MI-1 (DrMI-1) and *Syntrophobacter fumaroxidans* MPOB (SfMPOB)

S. FIG 5B: CLUSTAL 2.0.8 multiple sequence alignment: ApsB (DVU0846)

```
DvDP4           MPTYVDPSKCDGCKGGEKTACMYICPNDLMILDPEE------MRAYNQEPEACWECYSCV 54
DvH             MPTYVDPSKCDGCKGGEKTACMYICPNDLMILDPEE------MRAYNQEPEACWECYSCV 54
DdG20           MPTYVDPSKCDGCKGGEKTACMYICPNDLMILDPEE------MKAYNQEPEACWECYSCV 54
DpLSv54         MPSYVDPAKCDGCKGGDKTACMYICPNDLMVLDKVS------MKAYNQEPDGCWECYSCV 54
CtTLS           MPSFVIKEKCDGCKGQERTACMYICPNDLMKLDVER------MKAWNQEPDQCWECYNCV 54
CcCaD3          MPSFVIKEKCDGCKGQERTACMYICPNDLMKLDVDR------MVAWNQEPDQCWECYNCV 54
CpBS1           MPSFVIKEKCDGCKGQERTACMYICPNDLMKLDVDR------MLAWNQEPDQCWECYNCV 54
Td              MPTYVRTDKCDGCKGQDKTACMYICPHDLMKLDKDGSETGHAMRAFNQEPEQCWECYSCV 60
DrMI-1          MPSFVIAEKCDGCKGQDKTACMYICPNDLMVLDKEK------MKAYNRDTSQCWECYCCV 54
SfMPOB          MPSYVILEKCDGCKGQDKTACMYICPNDLMVLDKEK------MKAFNRAPEMCWECYNCV 54
Af              MPSFVNPEKCDGCKALERTACEYICPNDLMTLDKEK------MKAYNREPDMCWECYSCV 54
                **::*   ******. ::*** ****:*** **         * *:*: .. ***** **

DvDP4           KICPQGAITARPYADFAPMGGTCIPMRSADSIMWTVKFRNGNVKRFKFPIRTTPEGSIKP 114
DvH             KICPQGAITARPYADFAPMGGTCIPMRSADSIMWTVKFRNGNVKRFKFPIRTTPEGSIKP 114
DdG20           KICPQGAITARPYADFAPMGGTSIPMRSADSIMWTVKFRNGNVKRFKFPIRTTPEGSIKP 114
DpLSv54         KICPQGAIFVRGYDDFVPMGGQVHPMRSADSIMWTVKFRNGALKRFKFPIRTTAEGAANA 114
CtTLS           KICPQQAIEVRGYADFVPLGGNVIPLRGTDAIMWTIKFRNGILKRYKFPIRTTSEGSIDP 114
CcCaD3          KICPQQAIEVRGYADFVPLGGNVIPLRGTDAIMWTIKFRNGILKRYKFPIRTTAEGSIEP 114
CpBS1           KICPQQAIEVRGYADFVPLGGNVIPLRGTDAIMWTIKFRNGTLKRFKFPIRTTAEGSIEL 114
Td              KICPQQAIEARHYADVVPLGGMVQPLRGSDSIMWTIKFRNGTLKRFKFPIRTTPEGSIDP 120
DrMI-1          KICPQQAIDVRGYADFVPMGASCVPLRSSDSIMWTVKFRNGMLKRFKFPIRTTDEGTAVP 114
SfMPOB          KICPQQAIDVRAYADFVPMGASVVPLRGSEDIMWTVKFRNGMVKRFKFPIRTTPEGKADP 114
Af              KMCPQGAIDVRGYVDYSPLGGACVPMRGTSDIMWTVKYRNGKVLRFKFAIRTTPWGSIQP 114
                *:*** ** .* * *  *:*.   *:*.:. ****:*:*** : *:**.****  *

DvDP4           FEGKPEA--GDLESELLFTETALTAPKEALGKKFDVTDAETSQCWYETGCQGGNR 167
DvH             FEGKPEA--GDLESELLFTETALTAPKEALGKKFDVTDAETSQCWYETGCQGGNR 167
DdG20           FEGKPEP--GDLENELLFTETELGAPETALGQKFDVAEADKAVEFKASAV----- 162
DpLSv54         YVG-AAG--ASLDDERLLLEGELPTPK---------------------------- 138
CtTLS           YSGKPEPDYANLKKPGFFNMTEYPTL----------------------------- 140
CcCaD3          YAGKPEPDYANLKKPGFFNMTEYPTI----------------------------- 140
CpBS1           YSGKPEHDYDNLKKPGFFNMTEYPTL----------------------------- 140
Td              FGGKPTAKLADLTATGFFTGSADGGITKGYRPGNPAELIRK-------------- 161
DrMI-1          DGGYTLDQED-LNSQLLFTEPYSTGTPELPGLKK--------------------- 147
```

```
SfMPOB          LAGFATGTDD-IKSPVLCTEPASTWASELPTLKK--------------------- 147
Af              FEGFPEPTEEALKSELLAGEPEIIGTSEFPQVKKKA------------------- 150
                  * .      :    :
```

Note: Boxed region indicates position of lysine residue that was found to be methylated in ApsB (DVU0846). Homologs from the following organisms were used for this alignment: *Desulfovibrio vulgaris* DP4 (DvDP4), *Desulfovibrio vulgaris* Hildenborough (DvH), *Desulfovibrio desulfuricans* G20 (DdG20), *Desulfotalea psychrophila* LSv54 (DpLSv54), *Chlorobium tepidum* TLS (CtTLS), *Chlorobium chlorochromatii* CaD3 (CcCaD3), *Chlorobium phaeobacteroides* BS1 (CpBS1), *Thiobacillus denitrificans* (Td), *Desulfotomaculum reducens* MI-1 (DrMI-1), *Syntrophobacter fumaroxidans* MPOB (SfMPOB) and *Archaeoglobus fulgidus* (Af).

S. FIG 5C: CLUSTAL 2.0.8 multiple sequence alignment: DsrC (DVU2776)

```
DvDP4               MAEVTYKGKSFEVDEDGFLLR-FDDWCPEWVEYVKESEGISDISPDHQKIIDFLQDYYKK 59
DvH                 MAEVTYKGKSFEVDEDGFLLR-FDDWCPEWVEYVKESEGISDISPDHQKIIDFLQDYYKK 59
DdG20               MAEVSFQGKTFEVDEDGFLLR-FDEWCPEWVEYVKESEGIAEITEDHQKIIDFLQDYYRK 59
LiPHE-MN1-00        MAEVIYKGKNFEVDEDGFLLN-FENWGPEWVDYVKESEGISEVTDAHRKIIEFLQDYYKK 59
SfMPOB              MGQIEFKGKQFEVDEDGFISS-FDQWNPDWVEYVKTSEGITDLTEEHWKVIHVLQDYYKK 59
CDoHxd3             MATIEFQGKSFNVDEDGFIES-YSEFSEEWVQWVKKEEGIDELTDEHRQVIKVLQDYYEK 59
DpLSv54             MPTLEHNGSSFQVDEDGFLLTGMEEFNEDWIEYVSSVEGIEELTDEHRKVIDSLQEYYKK 60
TcNor1              MAFLNVNGKQIEIDEDGFLVN-PEDWNEEVVQAFAKLEDVAELTEDHWKVINYLRDYYKQ 59
MtATCC39073         MPTVNLAGMQLEVDEDGFIAD-PSKWNEDVARALAEQEGVTEMTEDHWKLVNYLRQYYLQ 59
DrMI-1              MAFIEINGQQFEVDEDGFLLN-PDTWIEDVAKYFCKEEGIDELTEDHWKVIKYLRDYFFQ 59
DaMP104C            MPFVEVEGKQVEVDEDGFMVD-PAAWWPGWAHYVAKQEGIGELTERHWHVIDFLKDYYAK 59
                    *  :   *  .::*****:      :       .   *.: :::  *:::. *::*: :

DvDP4               NGIAPMVRILSKNTGFKLKEVYELFPSGPGKGACKMAGLPKPTGCV 105
DvH                 NGIAPMVRILSKNTGFKLKEVYELFPSGPGKGACKMAGLPKPTGCV 105
DdG20               NGIAPMVRILSKNTGFKLKQVYELFPSGPGKGACKMAGLPKPTGCV 105
LiPHE-MN1-00        NGIAPMVRIISKNTGYKLKEIYEFFPSGPGKGACRMAGLPKPTGCV 105
SfMPOB              HGIAPMVRILTKVTGYKLKYIYELFPSGPGKGACKMAGLPKPTGCV 105
CDoHxd3             NGIAPMVRILSKLTGFKLKHIYELFPSGPGKGACKMAGLPKPTGCV 105
DpLSv54             NGIAPMVRILSKTTGFPLKRIYELFPSGPGKGACKMAGLPKPTGCV 106
TcNor1              FGIAPMIRKLCKDTGFTLKQIYDMFPSGPAKGACKLAGLPKPTGCV 105
MtATCC39073         FGIAPMIRKVCKETGFSLKQIYDLFPSGPAKGACKVAGLPKPTGCV 105
DrMI-1              FGIAPMVRKLCKDTGYSLKGIYELFPTGPAKGACKLAGLPKPTGCV 105
DaMP104C            TGIAPMIRKLCKESGIPLKEMYELFPTGPAKGACKCAGLPKPTGCV 105
                     *****:* : * :*  ** :*::**:**.****: **********
```

Note: Boxed regions indicate positions of lysine residues that were found to be methylated in DsrC (DVU2776). Homologs from the following organisms were used for this alignment: *Desulfovibrio vulgaris* DP4 (DvDP4), *Desulfovibrio vulgaris* Hildenborough (DvH), *Desulfovibrio desulfuricans* G20 (DdG20), *Lawsonia intracellularis* PHE/MN1-00 (LiPHE-MN1-00), *Syntrophobacter fumaroxidans* MPOB (SfMPOB), *Candidatus Desulfococcus oleovorans* Hxd3 (CDoHxd3), *Desulfotalea psychrophila* LSv54 (DpLSv54), *Thermosinus carboxydivorans* Nor1 (TcNor1), *Moorella thermoacetica* ATCC 39073 (MtATCC39073) *Desulfotomaculum reducens* MI-1 (DrMI-1), and *Desulforudis audaxviator* MP104C (DaMP104C).

S. FIG 5D: CLUSTAL 2.0.8 multiple sequence alignment: RplL (DVU2927)

```
DvH                MS-ITKEQVVEFIGNMTVLELSEFIKELEEKFGVSAAAPMAAMAVAAPAGDAAPAE-EEK 58
DvDP4              MS-ITKEQVVEFIGNMTVLELSEFIKELEEKFGVSAAAPMAAMAVAAPAGDAAPAE-EEK 58
DdG20              MSDITKEQVVEFIAGMTVLELSEFIKELEEKFGVSAAAPVAAMA-AAPAEAAAPAE-EEK 58
LiPHE-MN1-00       MS-VTKEQVIEFISNMTVLELSQFIKELEEKFGVSAAAPAVGMMMAAPTAASAEAEAEEK 59
GmGS-15            MAEITKADVVSFIENMTVLELSELVKELEEKFGVSAAAPVAVAAAAAPAAAAEAAE--EK 58
GsPCA              MAEITKADVISFIENMTVLELAELVKELEDKFGVSAAAPVAVAAAAAPAAAAEAAE--EK 58
Gsp.FRC-32         MAEITKADVISFIEKMSVLELAEMVKELEEKFGVSAAAPVAVAAVAGP-AAAEAAE--EK 57
GlSZ               MAEITKADVVAFIEKMTVLELAELVKELEEKFGVSAAAPVAVAA-AAPAAAAEAAE--EK 57
PpDSM2379          MAEITKQDVIEYIEKMSVLELSELVKELEEKFGVSAAAPVAVAAAGPATADAAAAE--EK 58
DaDSM684           MADITKEQVIEFIENMSVLELSELVKELEDKFGVSAAAPVAVAAAGPAGDAGAAAE--EK 58
Dspp.              MADITKEQVIEFIENMSVLELSELVKELEDKFGVSAAAPVAVAAAGPAGDAGAAAE--EK 58
                   *: :** :*: :*  *:****::::****:********* .    . .   .  **  **

DvH                TEFDIILKSAGANKIGVIKVVRALTGLGLKEAKDKVDGAPSTLKEAASKEEAEEAKKQLV 118
DvDP4              TEFDIILKSAGANKIGVIKVVRALTGLGLKEAKDKVDGAPSTLKEAASKEEAEEAKKQLV 118
DdG20              TEFDVILKSPGANKIAVIKVVRALTGLGLKEAKEKVDGTPSTLKEAVSKDDAEEAAKQLK 118
LiPHE-MN1-00       TEFDVILKEAGANKIAVIKVVRALTGLGLKEAKDKVDGAPSTLKEGVSKEDAEEAKKQLT 119
GmGS-15            TEFDIILKSAGANKIGVIKVVRALTGLGLKEAKDLVDGAPKSVKTGVSKEEAEDAKKQLV 118
GsPCA              TEFDVILKSAGANKIGVIKVVRALTGLGLKEAKDLVDGAPKPVKNGVSKEEAEEAKKQLV 118
Gsp.FRC-32         TEFDVILKAAGANKIGVIKVVRALTSLGLKEAKDLVDGAPQPLKTGVSKEEAEEAKKQLV 117
GlSZ               TEFDVILKSAGANKINVIKVVRTLTSLGLKEAKDLVDGAPSPVKTGISKAEAEEAQKQLV 117
PpDSM2379          TEFDVILKAAGANKINVIKVVRALTSLGLKEAKDLVDGAPGAVKTGVSKQEAEDAQKQLV 118
DaDSM684           TEFDVVLTGAGDKKINVIKVVRSVTGLGLKEAKEMVDGAPSTVKEAAAKEEAEDIKKQLE 118
Dspp.              TEFDVVLTGAGDKKINVIKVVRSVTGLGLKEAKEMVDGAPSTVKEAAAKEEAEDIKKQLE 118
                   ****::*. .* :** ******::*.*******: ***:* .:* . :* :**:  ***

DvH                EAGAEVEIK 127
DvDP4              EAGAEVEIK 127
DdG20              EAGAEVEVK 127
LiPHE-MN1-00       EAGATVEVK 128
GmGS-15            ESGAEVEIK 127
GsPCA              ESGAEVEIK 127
Gsp.FRC-32         EAGAEVEIK 126
GlSZ               EAGAEVEIK 126
PpDSM2379          EAGAEVEVK 127
```

```
DaDSM684            EAGASVELK 127
Dspp.               EAGASVELK 127
                    *:** **:*
```

Note: Boxed regions indicate positions of lysine residues that were found to be methylated in RplL (DVU2927). Homologs from the following organisms were used for this alignment: *Desulfovibrio vulgaris* DP4 (DvDP4), *Desulfovibrio vulgaris* Hildenborough (DvH), *Desulfovibrio desulfuricans* G20 (DdG20), *Lawsonia intracellularis* PHE/MN1-00 (LiPHE-MN1-00), *Geobacter metallireducens* GS-15 (GmGS-15), *Geobacter sulfurreducens* PCA (GsPCA), *Geobacter* sp. FRC-32 (Gsp.FRC-32), *Geobacter lovleyi* SZ (GlSZ), *Pelobacter propionicus* (DSM 2379), *Desulfuromonas acetoxidans* DSM 684 (DaDSM684) and *Desulfuromonas* spp. (Dspp.).
