# Supplementary material for: Towards a Rigorous Network of Protein-Protein Interactions of the Model Sulfate Reducer Desulfovibrio vulgaris Hildenborough
Source: PLoS One. 2011 Jun 28;6(6):e21470. doi: 10.1371/journal.pone.0021470 (PMC3125180; doi:10.1371/journal.pone.0021470)
Supplement: Figure S6 — Hidden Markov Model Alignments. Note: Boxed region indicates position of methylated lysine residue observed in this study. (PDF) [file pone.0021470.s006.pdf]

Figure S6: Hidden Markov Model Alignments

S. FIG 6A: Alignment of ApsB (DVU0846) with TIGR02060

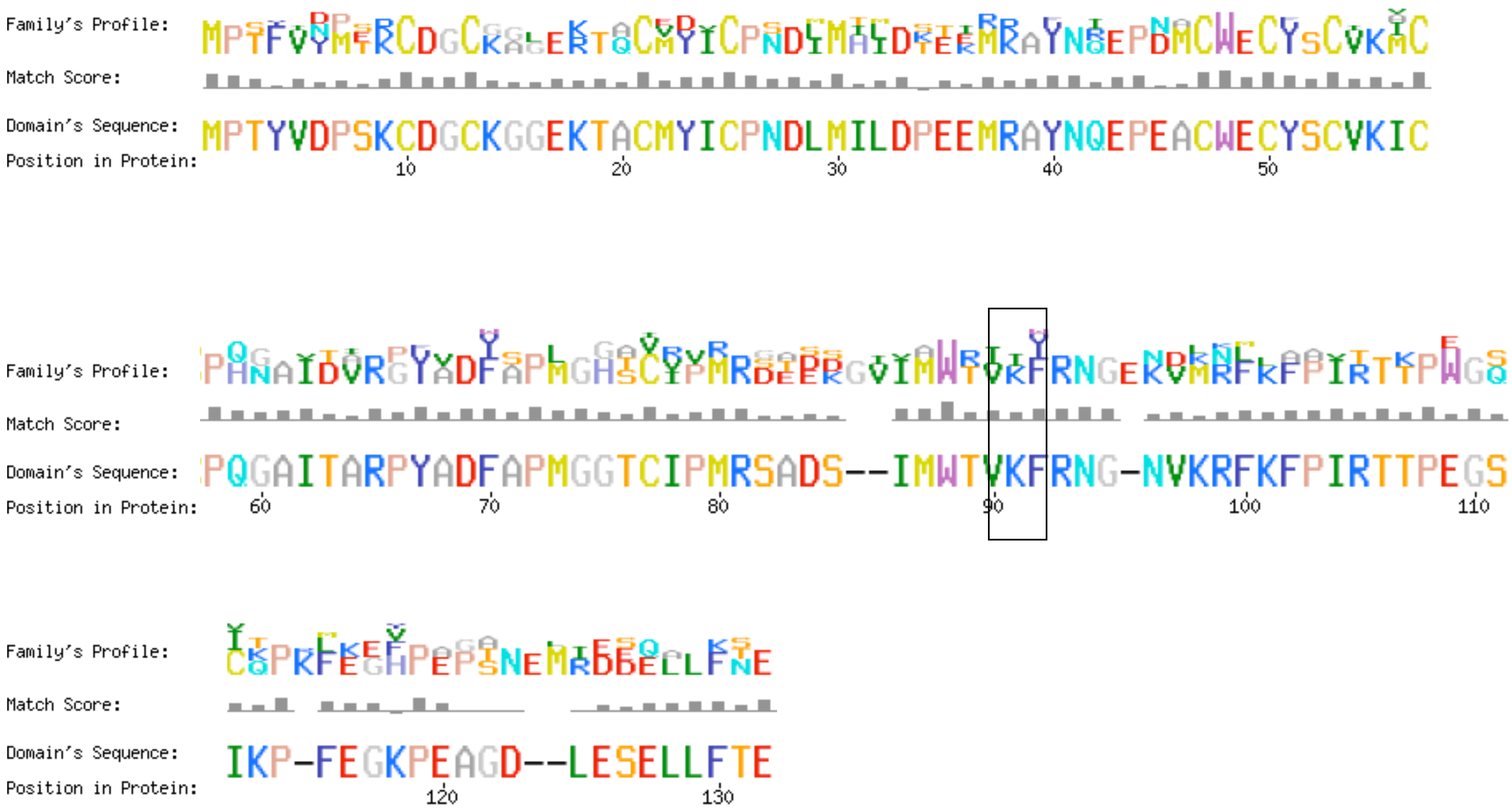

S. FIG 6B: Alignment of ApsA (DVU0847) with TIGR02061

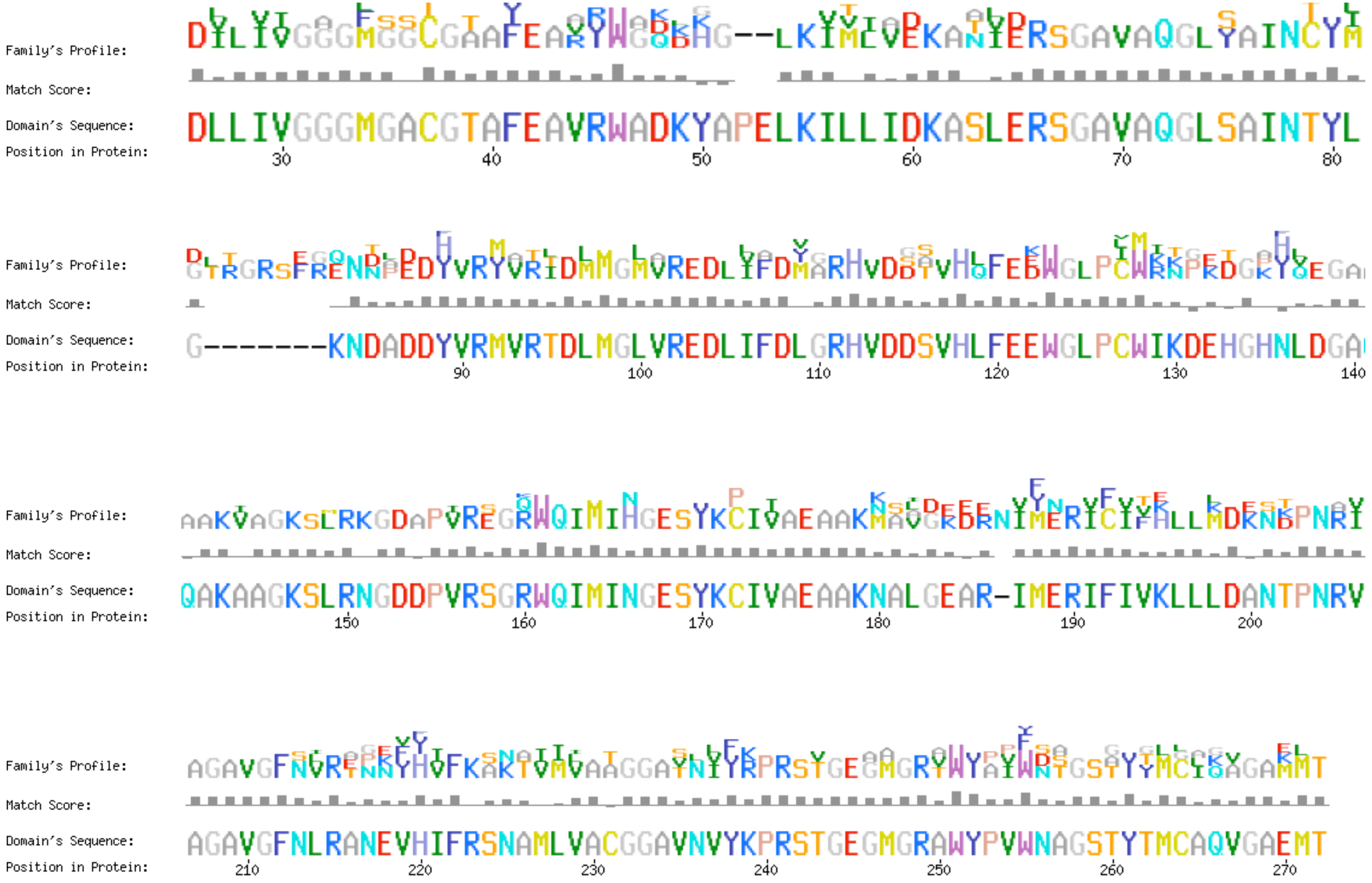

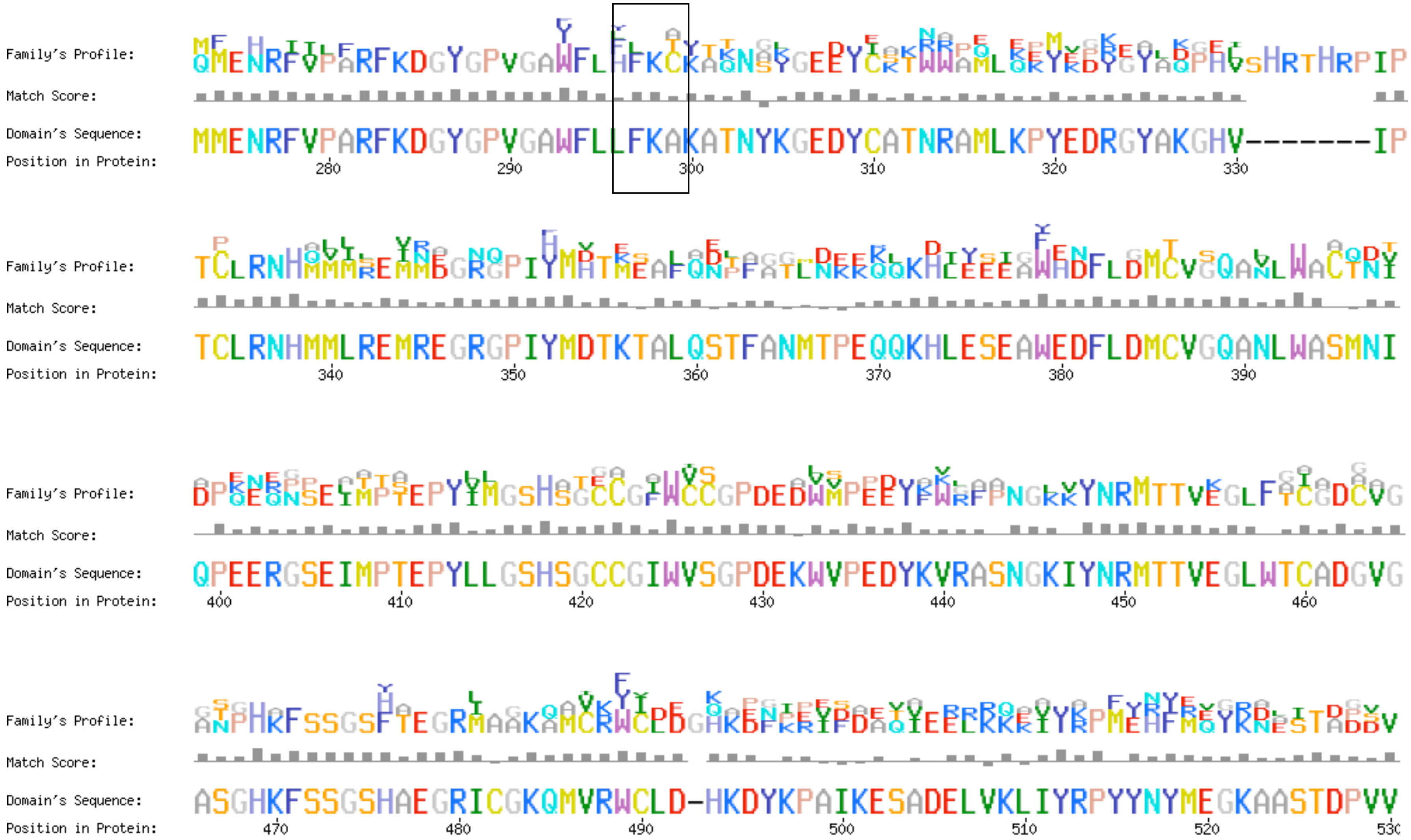
Family's Profile:
Match Score:
Domain's Sequence:
Position in Protein:
MMENRFVPARFKDGYGPVGAWFLLFKAKATNYKGEDYCATNRAMLKPYEDRGYAKGHV-------IP
280 290 300 310 320 330
TCLRNHMMLREMREGRGPIYMDTKTALQSTFANMTPEQQKHLESEAWEDFLDMCVGQANLWASMNI
340 350 360 370 380 390
QPEERGSEIMPTEPYLLGSHSGCCGIWVSGPDEKWVPEDYKVRASNGKIYNRMTTVEGLWTCADGVG
400 410 420 430 440 450 460
ASGHKFSSGSHAEGRICGKQMVRWCLD-HKDYKPAIKESADELVKLIYRPYYNYMEGKAASTDPVV
470 480 490 500 510 520 530

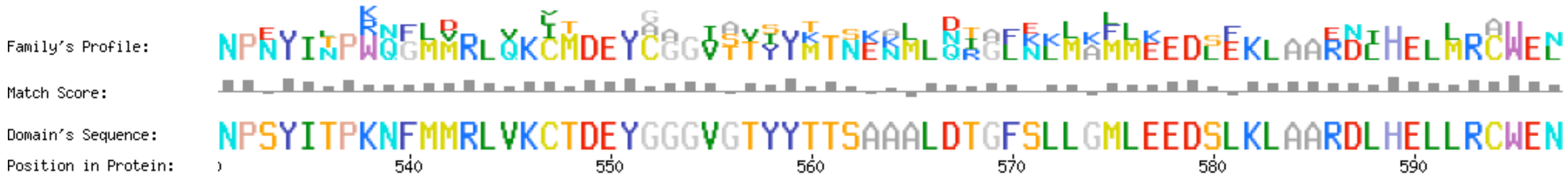
Family's Profile:
Match Score:
Domain's Sequence:
NPSYITPKNFMMRLVKCTDEYGGGVGTYYTTSAAALDTGFSLLGMLEEDSLKLAARDLHELLRCWEN
Position in Protein:
540
550
560
570
580
590

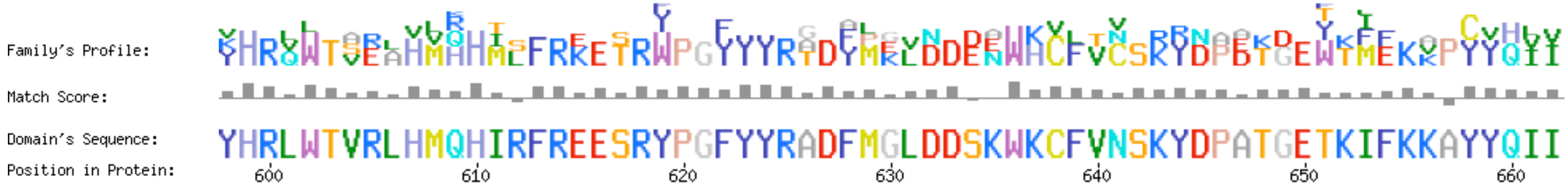
Family's Profile:
Match Score:
Domain's Sequence:
YHRLWTVRLHMQHIRFREESRYPGFYYRADFMGLDDSKWKCFVNSKYDPATGETKIFKKAYYQII
Position in Protein:
600
610
620
630
640
650
660

S. FIG 6C: Alignment of DsrC (DVU2776) with PF04358

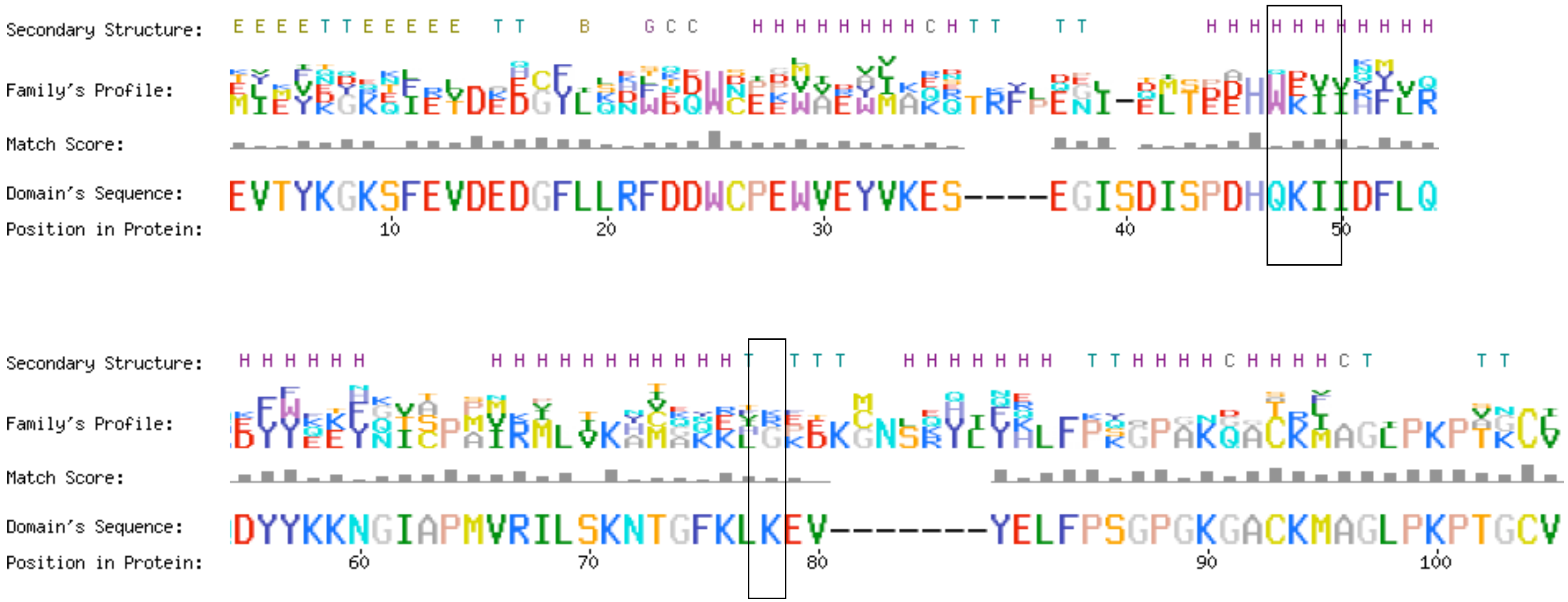

S. FIG 6D: Alignment of RplL (DVU2927) with TIGR0855

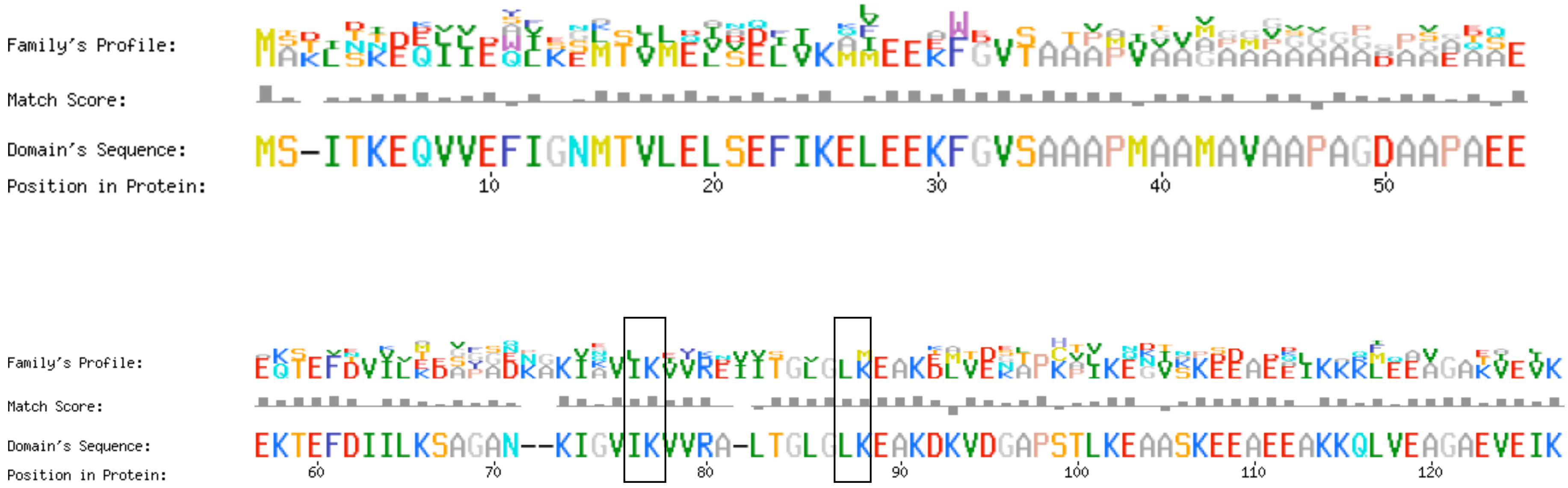

Note: Boxed region indicates position of methylated lysine residue observed in this study.
