# Supplementary figures and images for: Towards a Rigorous Network of Protein-Protein Interactions of the Model Sulfate Reducer Desulfovibrio vulgaris Hildenborough
Source: PLoS One. 2011 Jun 28;6(6):e21470. doi: 10.1371/journal.pone.0021470 (PMC3125180; doi:10.1371/journal.pone.0021470)

**Figure S7: Q-Star MS/MS data for ApsA, ApsB, and DsrC post-translational modifications.**

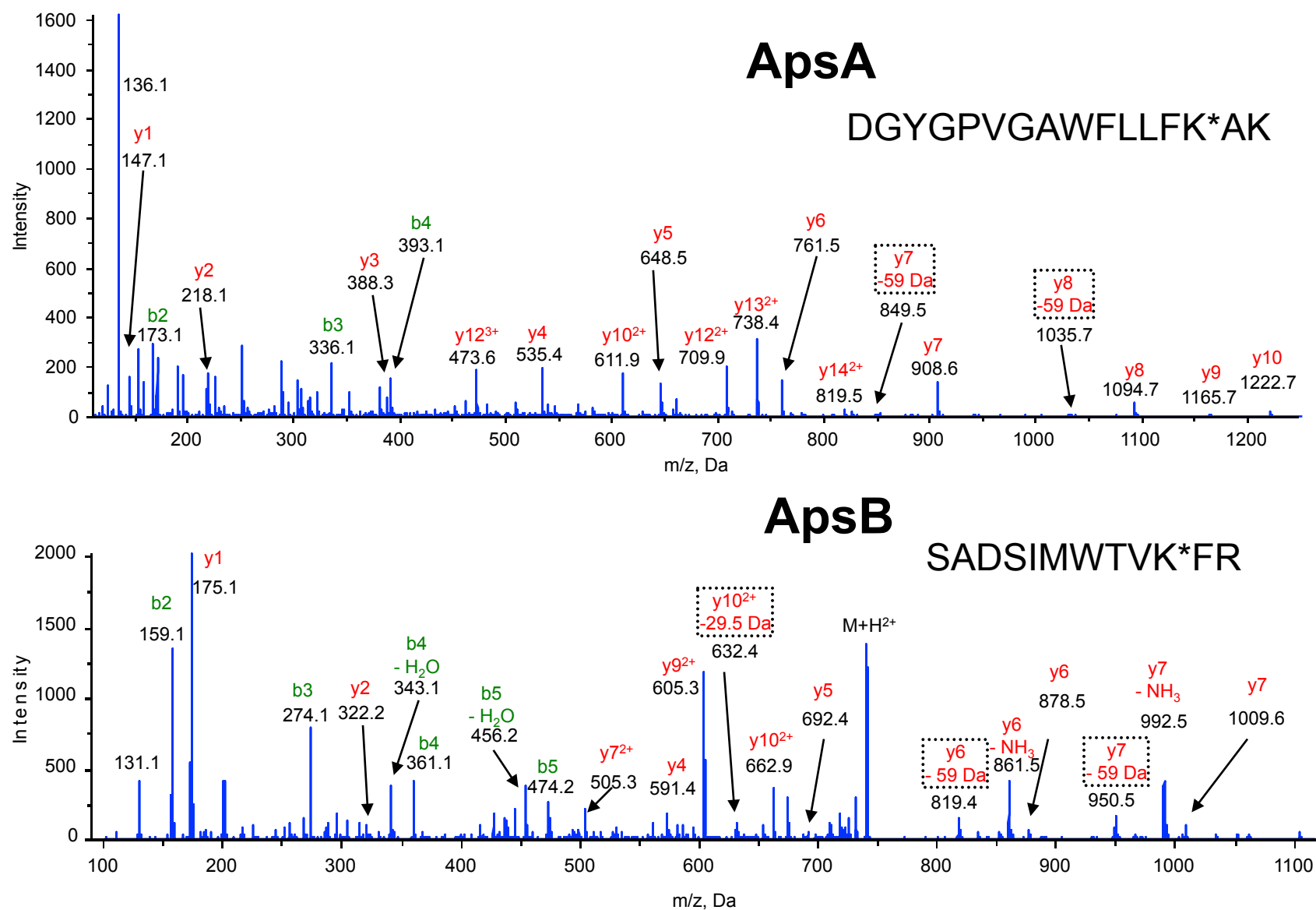

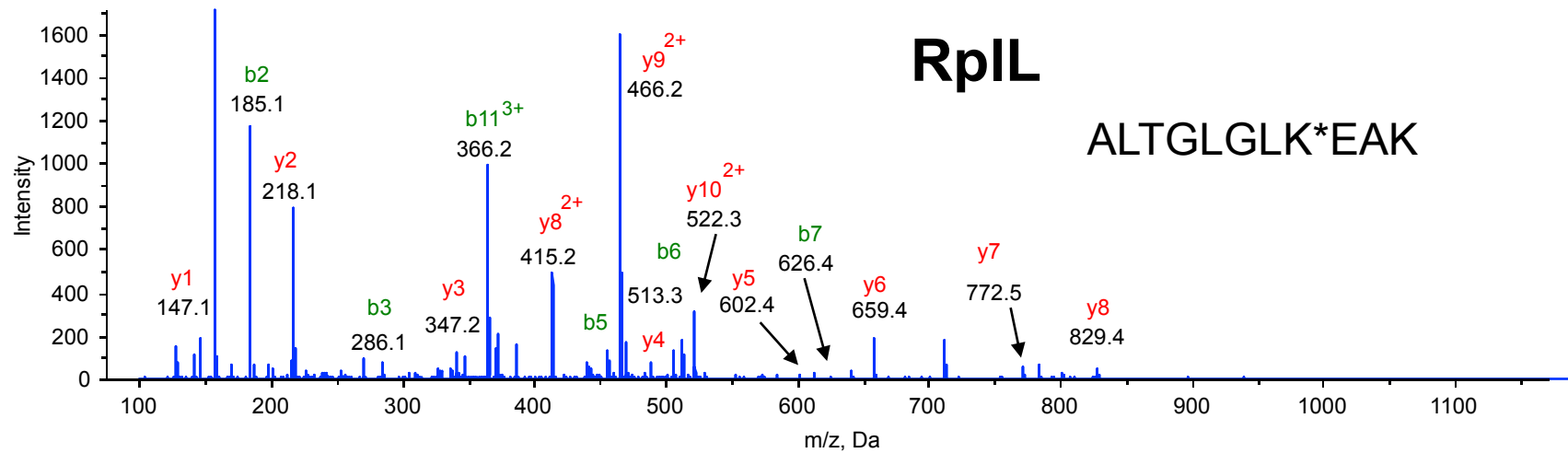

Supplement: Figure S7 — Q-Star MS/MS data for ApsA, ApsB, and DsrC post-translational modifications. (PDF) [file pone.0021470.s007.pdf]

**Fig S8:** Replicate agreement for pull-down experiments.

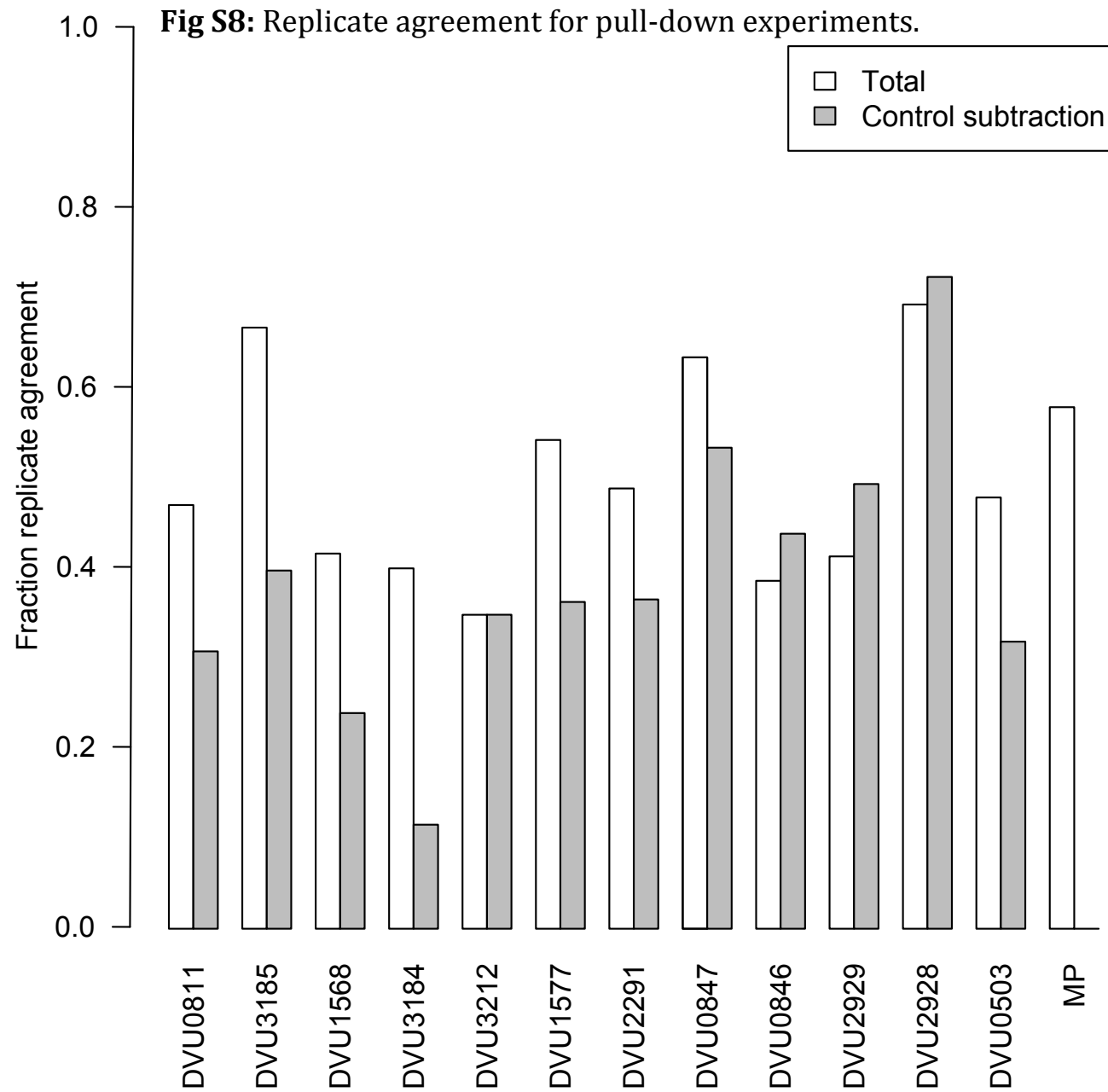

Supplement: Figure S8 — Replicate agreement for pull-down experiments. (PDF) [file pone.0021470.s008.pdf]
