# Supplementary material for: Towards a Rigorous Network of Protein-Protein Interactions of the Model Sulfate Reducer Desulfovibrio vulgaris Hildenborough
Source: PLoS One. 2011 Jun 28;6(6):e21470. doi: 10.1371/journal.pone.0021470 (PMC3125180; doi:10.1371/journal.pone.0021470)

Fig. S9. Cloning Schemes used for suicide vector construction

Scheme I

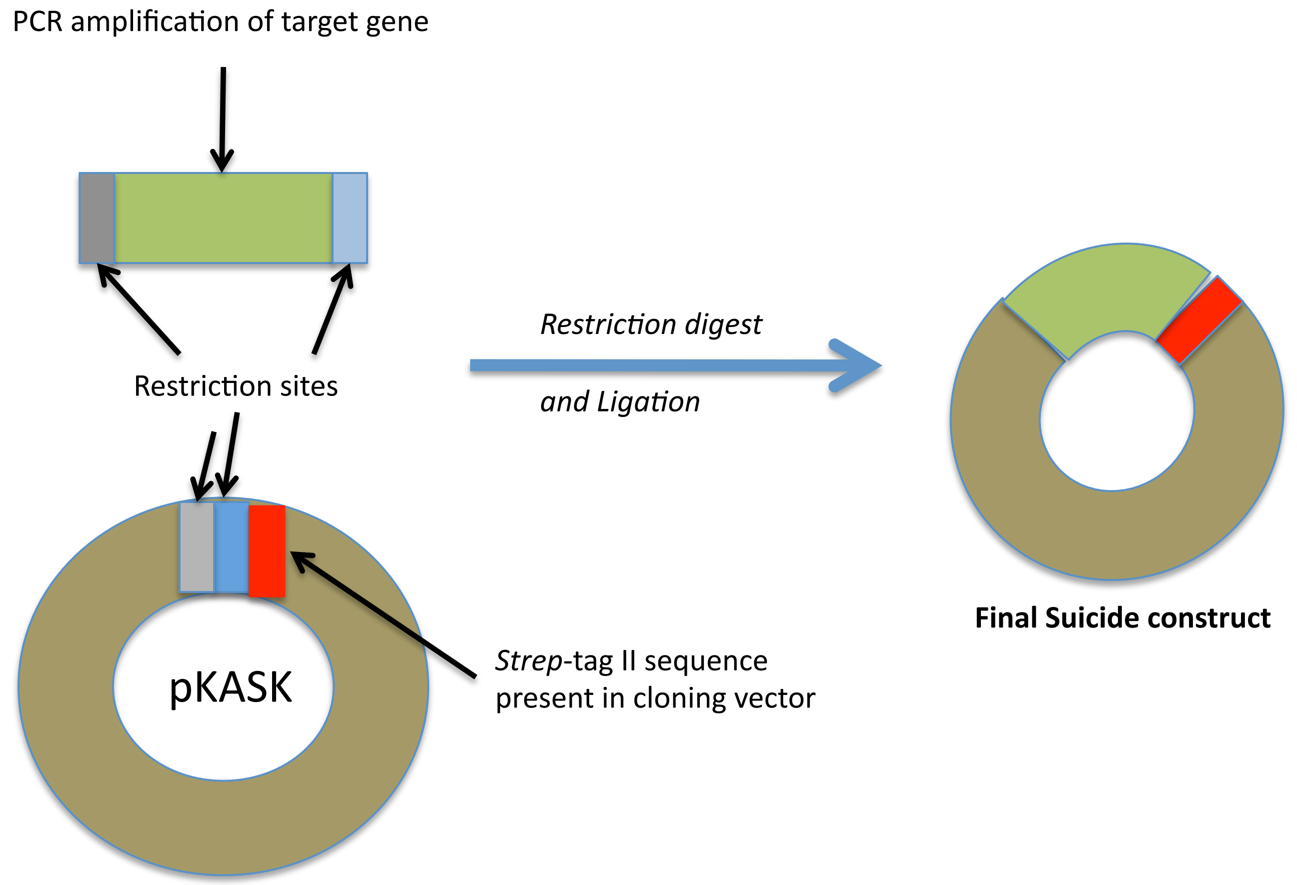

Scheme II

1. Orphan gene or last gene in operon (Green):

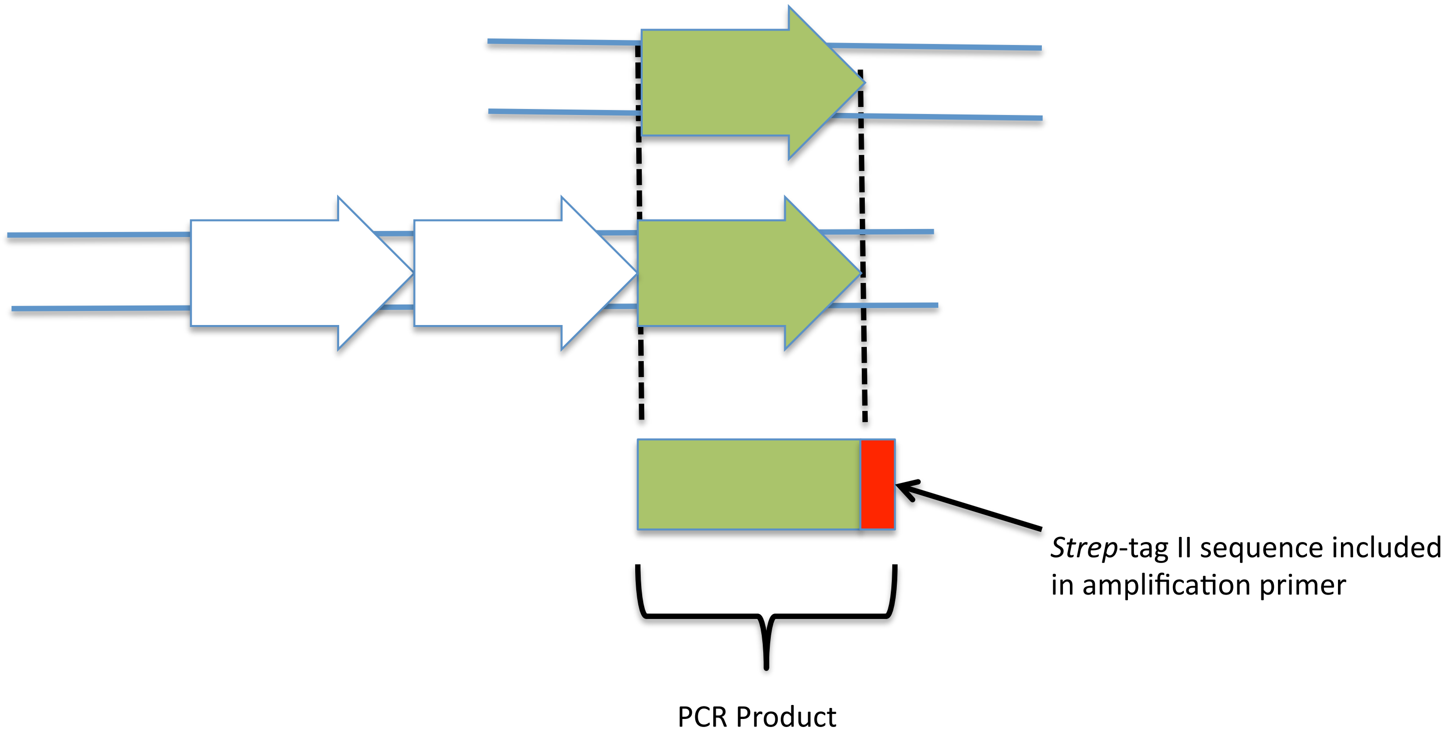

Scheme II

2. First or second gene in operon (Green):

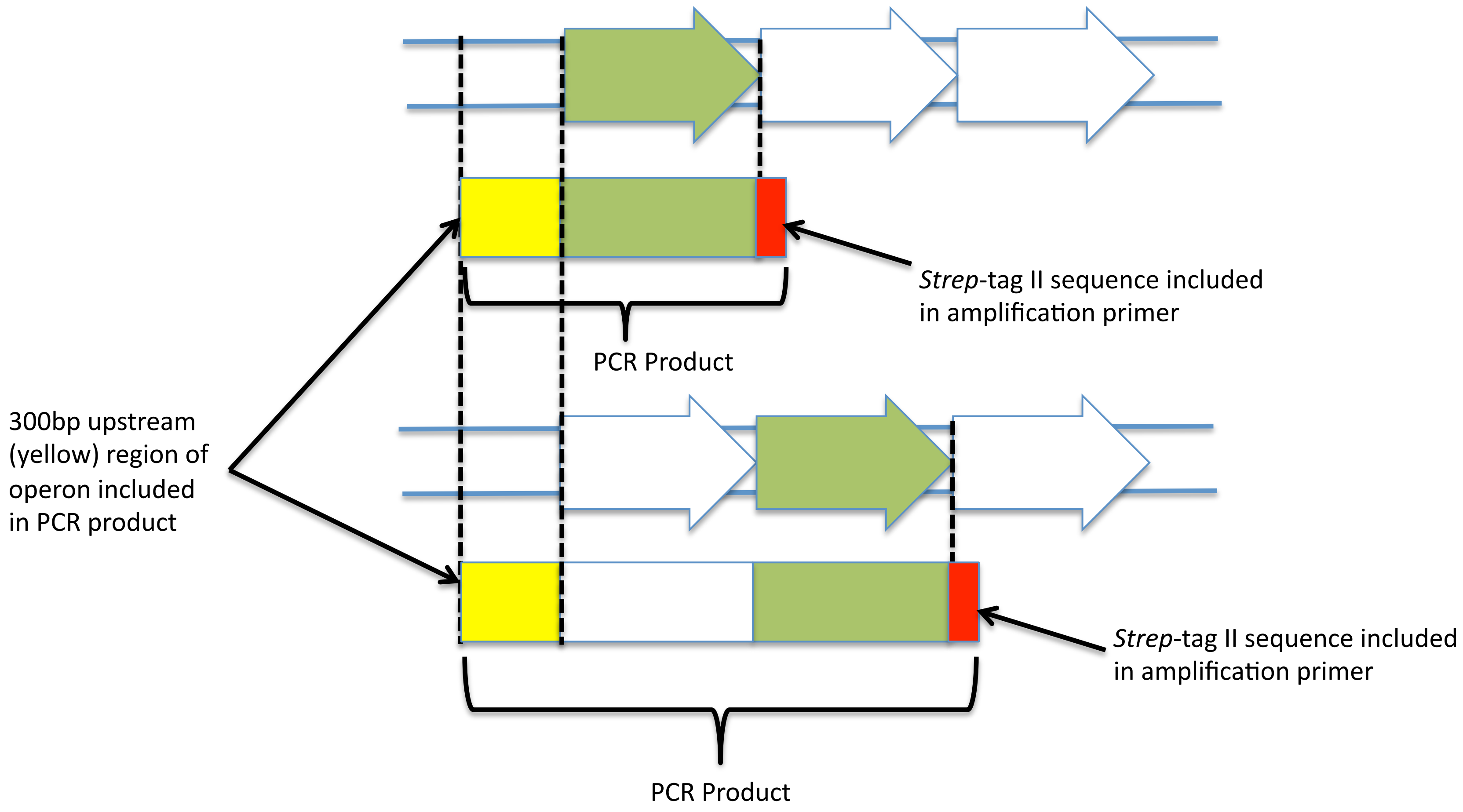

Scheme III

Used for *cooH* (DVU2291) and *rub* (DVU3184) (Green):

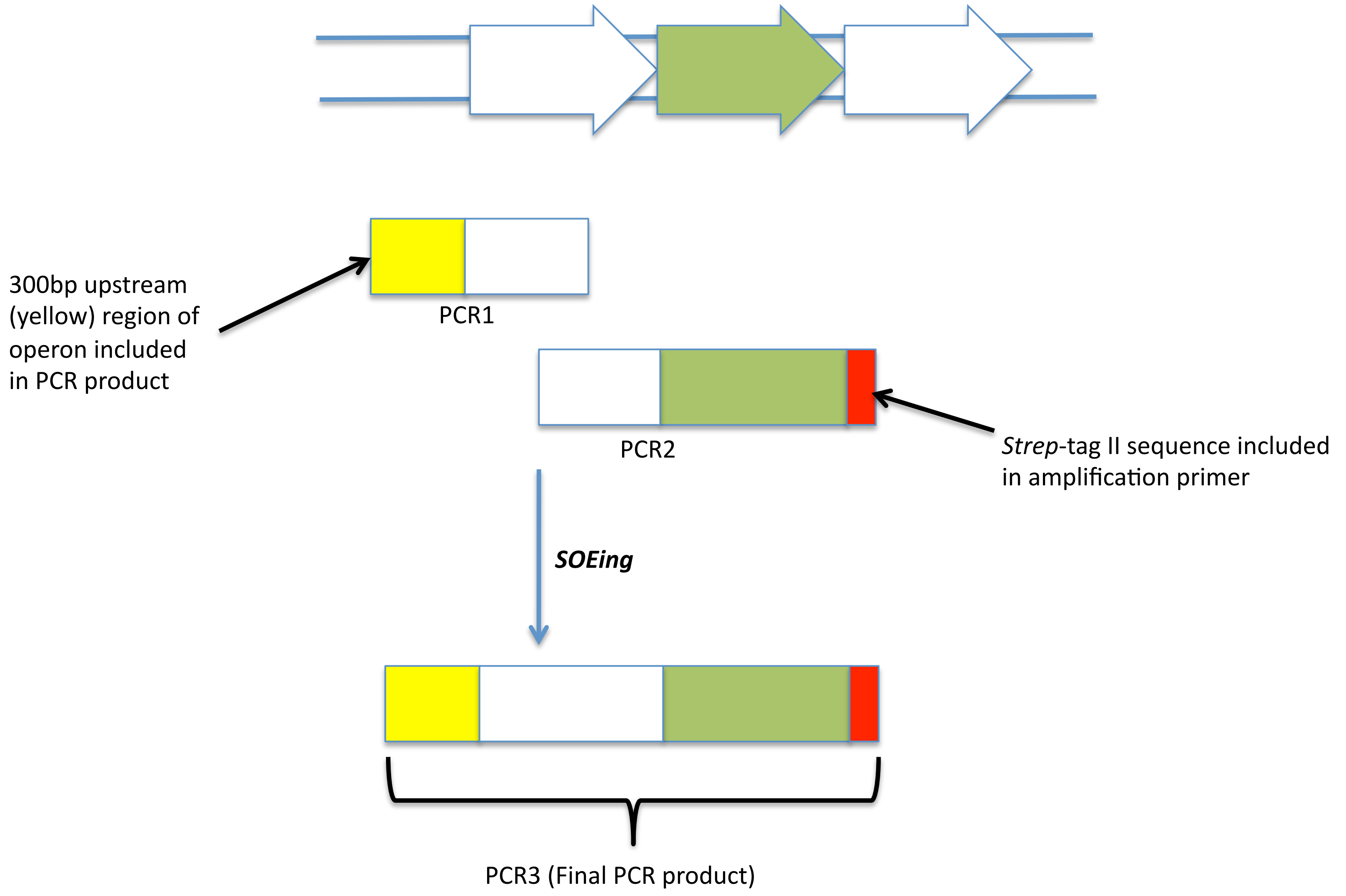

Supplement: Figure S9 — Cloning schemes used for suicide vector construction. (PDF) [file pone.0021470.s009.pdf]
