# Supplementary material for: Towards a Rigorous Network of Protein-Protein Interactions of the Model Sulfate Reducer Desulfovibrio vulgaris Hildenborough
Source: PLoS One. 2011 Jun 28;6(6):e21470. doi: 10.1371/journal.pone.0021470 (PMC3125180; doi:10.1371/journal.pone.0021470)
Supplement: Methods S1 — Supplementary Methods. (DOCX) [file pone.0021470.s024.docx]

**Supplementary Methods.**

***Transformation of JW801 strains.*** A 50 ml culture of *D. vulgaris* was grown to early- to mid-exponential phase (OD_600_ ca. 0.4) and collected by centrifugation (~4000g, 12 minutes). Cells were resuspended in an equal volume of chilled electroporation buffer (30 mM Tris) and harvested by a second round of centrifugation. The cell pellet was resuspended in 0.5 ml of electroporation buffer and 50 µl aliquots were placed into 0.5 ml centrifuge tubes. To each aliquot of cells, approximately 1 µg of plasmid DNA was added and the mixture transferred to an electroporation cuvette (1 mm gap, Molecular BioProducts, San Diego, CA). The cuvette was then subjected to electroporation at 1.75 kV, 250 W, and 25 µF with an ECM 630 (Harvard Apparatus, Holliston, MA). The cells were transferred into 1 ml of LS4 medium and allowed to recover overnight at 34 ^o^C. Three volumes of cells (10 µl, 100 µl, and the remaining volume) were used to inoculate molten LS4 medium containing G418, allowed to solidify, and incubated for 3-5 days at 34 ^o^C.

***Southern blots.*** In order to verify that plasmid integration occurred at the predicted location, a Southern blot was performed. Genomic DNA was prepared using the Wizard Genomic DNA Purification Kit (Promega, Fitchburg, WI) from 1.5 ml of culture grown anaerobically to early stationary phase in Wall LS3 medium. DNA was quantified with a ND-1000 spectrophotometer (Nanodrop, Wilmington, DE). Genomic DNA (2-5 μg) from wild-type cells and those with putatively tagged genes were digested at 37°C for 3h with 5-10 units of a restriction enzyme (New England Biolabs, Ipswich, MA or Promega, Fitchburg, WI) (Table S7). Restriction enzymes were chosen such that a single band would be visualized for the wild-type control DNA and two bands would be visualized for the DNA of the correctly integrated tagged construct when probed with the target gene. Separation of digested DNA was followed by transfer onto a Zeta-probe membrane (Bio-Rad, Hercules, CA). To the Zeta-probe membrane containing the digested genomic DNA, a probe was produced using the gene of interest as the template and the Primer-it II Random Primer Labeling Kit (Stratagene, Santa Clara, CA), per the manufacturers recommended instructions. The membrane was incubated for 30 - 60 min in hybridization solution (0.5 M sodium phosphate, 7% SDS, 1 mM EDTA) before the labeled-probe was added. The probe was hybridized to the blot overnight. The blot was washed with wash solution 1 (2xSSC and 0.1% SDS) for 30 min followed by a 30 min wash with wash solution 2 (0.1xSSC and 0.1%SDS). All incubations and washes were at 65^o^C. The blot was then exposed to x-ray film and the film developed. Band size was determined by comparison to the distance of its migration to those of the DNA fragments in the 1-Kb DNA ladder standard (NEB) as visualized on an agarose gel.

***Growth of JW801 strains and soluble protein extraction.*** Three one-liter cultures of each JW801 strain producing tagged proteins (Table S6) were grown anaerobically in LS4D medium containing G418 at 400 mg/ml [1,2]. Cells were harvested at late log phase (final optical densities are listed in Table S8). Briefly, the contents of the culture vessels were transferred to 50-ml prechilled (4°C) Falcon tubes and spun at 10,000 x g for 10 min at 4°C. The resulting supernatant was discarded, and the cell pellets were washed once with 100 mM Tris-HCl, pH 8.5 and stored at -80°C until analyzed. For soluble protein extraction, frozen cell pellets were resuspended in 100 mM Tris-HCl, pH 8.5 (2 ml), and lysed using a sonic dismembrator (Model #550; Fisher Scientific, Pittsburgh, PA). The cell lysate was centrifuged at 20,000 x g for 15 min at 4°C to separate the cell debris. The supernatant was subsequently used to determine the soluble protein profile of the cell. Protein samples were always maintained below 4°C, and a protease inhibitor cocktail consisting of EDTA-Na2 (0.5 mM), pepstatin (10 􏰆M), bestatin (0.13 mM), and Pefabloc SC plus (Roche Applied Science, Indianapolis, IN) (0.4 mM) was added to minimize degradation. Protein concentrations were deter- mined using the bicinchoninic acid protein assay (Pierce, Rockford, IL) with bovine serum albumin as the standard.

**References**

1. Chhabra SR, He Q, Huang KH, Gaucher SP, Alm EJ, et al. (2006) Global analysis of heat shock response in Desulfovibrio vulgaris Hildenborough. J Bacteriol 188: 1817-1828.

2. Mukhopadhyay A, He Z, Alm EJ, Arkin AP, Baidoo EE, et al. (2006) Salt stress in Desulfovibrio vulgaris Hildenborough: an integrated genomics approach. J Bacteriol 188: 4068-4078.
