# Supplementary material for: Towards a Rigorous Network of Protein-Protein Interactions of the Model Sulfate Reducer Desulfovibrio vulgaris Hildenborough
Source: PLoS One. 2011 Jun 28;6(6):e21470. doi: 10.1371/journal.pone.0021470 (PMC3125180; doi:10.1371/journal.pone.0021470)

Figure S2: Interactions in *E. coli* K12 (Hu *et al.*, 2009 and Butland *et al.*, 2005) between proteins that have orthologs in *D. vulgaris* and were detected in this study.

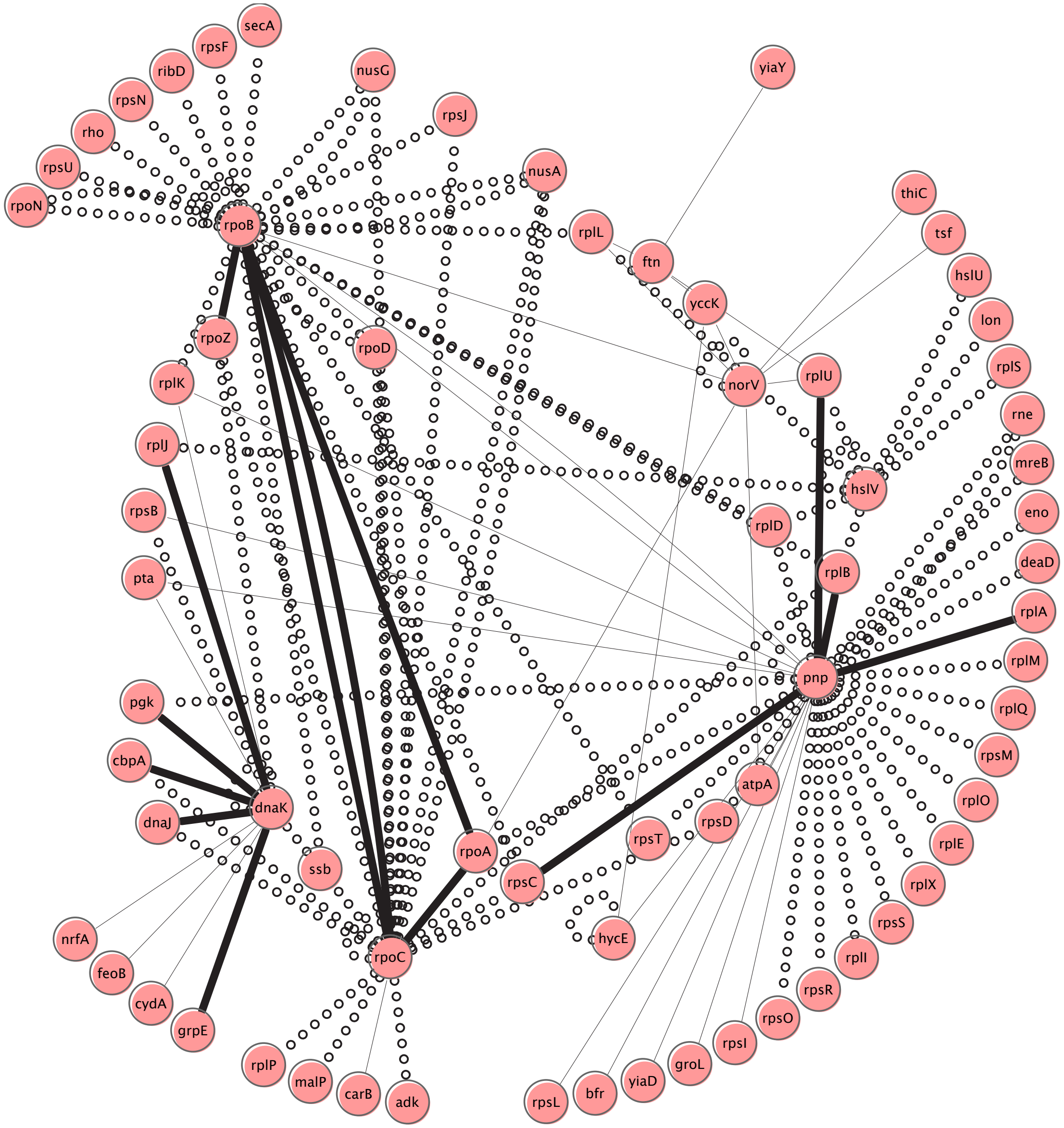

Supplement: Figure S2 — Interactions in E. coli K12 [11], [14] and D. vulgaris between proteins that have D. vulgaris orthologs in E. coli and which involve the D. vulgaris bait proteins used in this study. The edge thickness corresponds to protein interaction confidence in E. coli (arbitrarily 50 if only observed in Butland et al., otherwise confidence from Hu et al.). Black dotted edges indicate interactions observed in E. coli but not in D. vulgaris. Thick black solid edges indicate interactions observed in E. coli and in D. vulgaris. Thin solid edges indicate interactions detected in D. vulgaris but not E. coli. (PDF) [file pone.0021470.s002.pdf]
